# Alleviating the drought stress and improving the plant resistance properties of *Triticum aestivum via* biopriming with *aspergillus fumigatus*

**DOI:** 10.1186/s12870-024-04840-z

**Published:** 2024-02-28

**Authors:** Nelly Michel George, Gehad Hany-Ali, Ekram Abdelhaliem, Mohamed Abdel-Haleem

**Affiliations:** Department of Botany and Microbiology, Faculty of Science, Zagazig University, Zagazig, 44519 Egypt

**Keywords:** *Triticum aestivum*, Biopriming, Morphological, Physiological, Drought stress, *Aspergillus Fumigatus*, Gene expression

## Abstract

**Background:**

Wheat (*Triticum aestivum* L.) is one of the most widely grown and vital cereal crops, containing a high percentage of basic nutrients such as carbohydrates and proteins. Drought stress is one of the most significant limitations on wheat productivity. Due to climate change influences plant development and growth, physiological processes, grain quality, and yield. Drought stress has elicited a wide range of plant responses, namely physiological and molecular adaptations. Biopriming is one of the recent attempts to combat drought stress. Mitigating the harmful impact of abiotic stresses on crops by deploying extreme-habitat-adapted symbiotic microbes. The purpose of this study was to see how biopriming Triticum aestivum grains affected the effects of inoculating endophytic fungi *Aspergillus fumigatus* ON307213 isolated from stressed wheat plants in four model agricultural plants (Gemmiza-7, Sids-1, Sakha8, and Giza 168). And its viability in reducing drought stress through the use of phenotypic parameters such as root and shoot fresh and dry weight, shoot and root length, and so on. On a biochemical and physiological level, enzymatic parameters such as catalase and superoxidase dismutase are used. Total phenolics, flavonoids, and photosynthetic pigments are non-enzymatic parameters. Making use of molecular techniques such as reverse transcriptase polymerase chain reaction (RT-PCR).

**Results:**

It has been found that using *Aspergillus fumigatus* as a biological biopriming tool can positively impact wheat plants experiencing drought stress. The total biomass of stressed wheat plants that had been bio-primed rose by more than 40% as compared to wheat plants that had not been bio-primed. *A. fumigatus* biopriming either increased or decreased the amount of enzymatic and non-enzymatic substances on biochemical scales, aside from the noticeable increase in photosynthetic pigment that occurs in plants that have been bio-primed and stressed. Drought-resistant genes show a biopriming influence in gene expression.

**Conclusions:**

This is the first paper to describe the practicality of *a. fumigatus* biopriming and its effect on minimizing the degrading effects of drought through water limitation. It suggests the potential applications of arid habitat-adapted endophytes in agricultural systems.

**Supplementary Information:**

The online version contains supplementary material available at 10.1186/s12870-024-04840-z.

## Introduction

*Triticum aestivum* (Bread wheat) is one of the key crops planted on 216.7 million hectares globally. Bread wheat is a high-protein cereal grain that provides 20% of total calories, enough to feed 30% of the world’s population, and is an excellent source of vitamins (E, B1, B2, and B3) and minerals (Mn, P, Cu, and Se) [1]. In recent years, there has been discussion about the importance of increasing wheat yield under abiotic stress conditions. Drought is a common environmental stress that significantly impacts plant development and growth, particularly in Egypt [2]. So, screening and development of specific genotypes of wheat to tolerate and resist environmental stresses is the challenge [2]. Drought drastically reduces flag leaf area, grain yield, and wheat productivity [3, 4]. Drought causes oxidative damage, decreases CO_2_ availability, and inhibits photosynthesis and respiration via reactive oxygen species (ROS) damage [5]. ROS are unfavorable byproducts of environmental stress-induced biochemical changes in plants. However, ROS can induce oxidative stress by raising particular types of ROS involving H_2_O_2_, O_2_, and OH^-^ [6]. In chloroplasts, H_2_O_2_ toxicity is particularly pronounced, where even low gas concentrations can disrupt active Calvin cycle enzymes and prevent photosynthetic carbon dioxide assimilation. Understanding the established genetic variability required for developing wheat varieties with desirable traits [7]. Drought is a major threat to plants as it harms their photic systems II (PS II), which are sensitive to environmental factors that cause tension and damage under dry conditions. This damage affects the metabolic processes of cells, especially photosynthesis and respiration. In fact, drought is one of the most critical factors that affect photosynthetic systems and can lead to changes in plant growth and development [8]. Plant height, leaf area, leaf breadth, stem density, shoot fresh weight, shoot dry weight, root fresh weight, root dry weight, and the number of leaves and fibrous roots are all affected by drought [9]. Furthermore, cultivars with a higher drought sensitivity showed a significantly higher decrease in the chlorophyll a/b ratio, whereas the reported decrease was insignificant in more drought-resistant cultivars [10]. Plants’ responses to oxidative stress are usually via activating a strong defensive line capable of neutralizing the free radicals, that could be using enzymatic and/or non-enzymatic [11]. Although these mechanisms have been partially specified, their activities depend on the species or genotypes, type, and stress intensity due to the complexity of their processes [12]. Several strategies have been implemented to overcome the hazardous effects of drought on wheat. Biopriming is one of the plants’ most well-known methods for reducing drought stress [13]. Hydropriming, osmo-priming, hormone priming, solid matrix, thermos-priming, nutria-priming, chemo-priming, and biological priming were all documented methods of priming [14]. Plant growth promotor microorganisms (PGPMs) can promote plant resilience through both direct and indirect processes. These microorganisms produce plant hormones and improve nutrient uptake via phosphate or potassium solubilization or nitrogen fixation [15]. Endophytic fungi help plants survive by interacting with many plant species and alleviating the effects of salt, drought, heat, herbivores, diseases, and nutrient limitations [16, 17]. Endophytes within plants activate stress response systems and produce anti-stress compounds such as phytohormones, which promote root growth and nutrient uptake. They also produce osmolytes for antioxidative defense and reduce the production of the stress hormone ethylene by increasing the enzyme 1-aminocyclopropane-1-carboxylate deaminase (ACC) [18]. The research’s objective was to evaluate wheat’s resistance to drought stress under biopriming conditions with the endophytic fungal isolates occupying the same plant. This evaluation is based on the plant’s morphological, metabolic, and molecular characteristics. *Aspergillus fumigatus* demonstrated its ability to alleviate the effects of drought stress.

## Materials and methods

### Plant material and growing conditions

The Wheat Research Department of the Field Crops Research Institute, Agricultural Research Center, Giza, Egypt, provided wheat grains of four different varieties, namely Sakha-8 (Sa), Sids-1 (Si), Giza-168 (Gi), and Gemmiza-7 (Ge). Table 1 shows detailed information about these varieties. After 14 days of germination, the wheat grains were planted in pre-sterilized soil in plastic pots with five plants per pot. The plants were watered twice a week with tap water [19]. In this experiment, plastic pots with dimensions of 23 by 17 centimeters were filled with 3 kg of air-dried soil that had been disinfected with 48% formalin. Wheat grains were added to the soil. On the 16th day, drought stress was applied to the plants. The pots were divided into five groups. One group was irrigated with one liter of tap water and served as the control group. The other four groups were subjected to drought conditions by withholding water up to 800 ml, 600 ml, 400 ml, and 200 ml, respectively, based on the pot’s water-holding capacity [20] until day 35. The plants were analyzed using ANOVA every three days, starting on day 16 and ending on day 35. The maximum concentration of drought that the wheat plants could tolerate was determined to be 200 ml. Endophytic fungal isolation was performed at that level. Samples were gathered from each variety and used for morphological determination. Additionally, some leaves were collected and stored in a deep freezer at -40 °C for physiological and genetic analyses [21].

**Table 1:** Origin, pedigree, and selection history of the four varieties of wheat and lines used in the present study

No.	Genotype	Origin	Pedigree and/or selection history
1	Sakha-8 (Sa)	Egypt	INDS/NORTENO PK3418-65-0 S-0 S
2	Sids-1 (Si)	Egypt	HD2172/PAVON//1158.57/MAYA74 SD46-4SD-2SD-1SD-0SD
3	Giza-168 (Gi)	Egypt	MRL/BUC//SERI CM93046-8 M-0Y-0 M-2Y-0B-0GZ
4	Gemmiza-7 (Ge)	Egypt	CMH74A.630/5x//Seri82/3/Agent CGM 4611-2GM-3GM-1GM-OGM

### Isolation of the endophytic fungi

The wheat leaves were cut into small cubes and treated to remove any surface contaminants. To do this, they were washed with 70% ethanol for 2 min, then with 2.5% sodium hypochlorite for another 2 min, and then rinsed with sterile distilled water. The sterilized plant parts were then placed on top of potato dextrose agar (PDA) with ampicillin (1 mg/mL) [16]. The endophytic fungal colonies were allowed to incubate for 7 days at a temperature of 30 °C. The developed hyphae were then purified by subculturing on PDA and stored as slope and plate cultures at 4 °C. Control plate media was used to ensure the sterility of the working area, while positive control of non-sterilized plant parts was used to check for any epiphytic fungal flora. Finally, the isolated endophytic fungi were identified. According to reference keys, the fungal isolates were identified to their species level by growing on PDA media for 10 days at 30 ºC [22]. Based on their rRNA sequence, the fungal isolates were identified using molecular methods [16]. The genomic DNA (gDNA) of the fungus was extracted and utilized as a template in PCR [23], for amplification of the internal transcribed spacer (ITS) flanking the 5.8 S region [16], with the primer set ITS4 5′-GGAAGTAAAAGTCGTAACAAGG-3′ and ITS5 5′-TCCTCCGCTTATTGA TATGC-3′. To perform PCR, we used a PCR master mixture called i-TaqTM (Cat. No. 25,027, INTRON Biotech). The reaction mixture was made by adding 10 µl of the PCR master mixture, 2 µl of fungal gDNA, 1 µl each of forward and reverse primers (10 pmol/l), and sterile distilled water to a final volume of 20 µl. The PCR conditions were set as described previously [24]. DNA ladder (1 kb Nex-gene Ladder, Puregene, Cat. # PG010-55DI) was used, and the amplicon was visualized using a gel documentation system. The ITS sequence was obtained and searched against non-redundant sequences in the National Center for Biotechnology Information (NCBI) database using Basic local alignment search tool (BLAST).

### Fungal deposition

#### *Aspergillus Fumigatus* was deposited into the GenBank

##### Fungal inoculation and grain biopriming condition

The fungal isolate was cultured in potato dextrose broth (PDA) at 30 °C for ten days [25]. The biomass was then collected and thoroughly washed with a sterile saline solution. The study was performed in the Zagazig University, Cairo, Egypt greenhouse. The mycelia were powdered using a mechanical mortar and used for biopriming experiments with wheat grains after being dried overnight at 40 °C (Fig. S2). The treated grains were surface sterilized and placed in a heap, covered with a moist jute sack for 48 h at 28 °C with 1% carboxymethylcellulose as an adhesive agent. The heap was kept under moist conditions until pre-radical emergence [26]. For the experiment, each wheat variety was subjected to two conditions - fungi-inoculated and non-inoculated, in both control and 200 ml. The pots used in the experiment contained 3 kg of air-dried soil which was disinfected with 4% formalin (Fig. S3). After the experiment was completed, which was on the 35th day, leaf samples were collected to estimate various growth and molecular parameters.

#### Determination of various plant growth parameters

##### Morphological parameters

The study involved analyzing 12 quantitative morphological traits along with the presence or absence of certain characteristics before and after fungi inoculation. The species description was used to estimate the standard deviation of each quantitative character’s average value, and the current character’s qualitative state was documented. These findings were documented in reference [27].

##### Biochemical assay

The wheat leaves were dehydrated in a vacuum oven at 45 °C and then powdered. A 10 g quantity of the powdered sample was mixed with 100 mL of 50% ethanol (at a ratio of 1:10, w/v) and stirred for 3 h at room temperature. The solvent within the filtrate was retrieved by using a rotary evaporator [28].

##### Total phenolic compounds (TPC)

The study aimed to determine the total phenolic compounds present in the plants. To do so, 50 µL of each sample (100 µg/mL) was mixed with 50 µl of diluted Folin-Ciocalteu reagent and 50 µL of 7.5% Na_2_CO_3_ in microtiter plate wells. The mixture was kept at room temperature for 60 min and was then evaluated at 760 nm with a microtiter plate reader (Biotech Elx808, USA). The overall polyphenol content was expressed as mg gallic acid equivalent/mL of the solution using the following linear equation:

$${\rm{y }} = {\rm{ }}0.005{\rm{x }} + {\rm{ }}0.1455,{\rm{ R}^{2}}{\rm{ }} = {\rm{ }}0.9957$$

##### The total flavonoid content (TF)

TF was calculated using a slightly modified method from previous studies. To do this, 20 µl of each solution (100 g/mL) were mixed with 20 µl of sodium nitrite (5%) in microtiter plate wells. The TF was then calculated as mg of quercetin equivalent per mL of each solution using the following linear formula:

$${\rm{y }} = {\rm{ }}0.0053{\rm{x }} - {\rm{ }}0.0022,{\rm{ R}^{2}}{\rm{ }} = {\rm{ }}0.9962$$

##### Proline and total protein content

The contents of leaf proline and total protein were determined using the rapid colorimetric assay, as recommended by [29].

##### Antioxidant assay

The radical scavenging activity of Sids-1, Giza168, Gemmiza-7, and Shakha8 and WPAgNPs at a concentration of (100 g/mL) was determined using the 2,2-diphenyl-1-picrylhydrazyl (DPPH) radical scavenging assay, with certain modifications based on [30].

Catalase: The activity of catalase activity was measured by Biodiagnostic, Kit No. CA 25 17, Egypt [31, 32].

Glutathione peroxidase: The activity of glutathione peroxidase was measured by utilizing Biodiagnostic, Kit No. GP 25 24 [33].

Superoxide dismutase: The activity of superoxide dismutase was estimated by Biodiagnostic, Kit No. SD 25 21, Egypt, as per [34] approach.

The pigment contents were measured by estimating the absorbance of chlorophyll a (Chl a), chlorophyll b (Chl b), and carotenoids (Car) at 663.8 nm, 646.8 nm, and 470 nm, respectively [35].

##### RNA isolation and Gene expression

The RNA extraction process was carried out on wheat leaves from different varieties, following the instructions provided by the manufacturer with some modifications as described by [36]. After extraction, the RNA was stored at -80 °C for later analysis. To protect RNA from degradation, a double volume (1 ml) of the RNA protects Reagent (Qiagen, Germany, GmbH) was added to one volume (0.5 ml) of the broth of the harvested plant. The mix was then vortexed and incubated for 5 min at room temperature, then centrifuged for 10 min. at 8000 rpm. The supernatant was decanted. Then 200 µl of TE buffer containing 1 mg/ml Lysozyme (Biochemica, Applichem) was added to the pellet. Also, 700 µl RLT buffer containing 10 µl β-mercaptoethanol per 1 ml was added. Then 500 µl of 100% ethanol was added, and the steps were completed according to the QIAamp RNeasy Mini kit (Qiagen, Germany, GmbH). N.B. On-column DNase digestion was done to remove residual DNA. Oligonucleotide Primers. Primers used were supplied from Metabion (Germany). To check the purity of the extracted RNA, electrophoresis was performed on 1.2% agarose gels, which were then stained with ethidium bromide (0.1 g/mL). The purity of RNA was evaluated using a nanodrop spectrophotometer (Thermo Scientific NanoDropTM 1000) to measure the absorbance ratios (A260/280) and (A260/230). The equivalent total RNA concentration from each sample was 10 ng/µl.

##### Complementary-DNA synthesis (cDNA)

To create complementary DNA (cDNA), we followed this method: We mixed 2 µg of template RNA, 1 µl of oligonucleotides, and up to 13.5 µl of nuclease-free water and incubated it for 5 min at 65 °C, then chilled it on ice according to the instructions from the ABT H-minus cDNA synthesis Kit manufacturer. We then incubated each reaction at 42 °C for 60 min, using a combination of 4 µl of 5X first strand buffer, 0.5 µl of H minus MMLV (200 unit/µl), and 2 µl of the dNTPs mixture (10mM). To stop the reactions, we heated them for 5 min at 70 °C. We obtained oligonucleotide primers from Macrogen Company to detect the effect of abiotic stress and biopriming on wheat plants (see supplementary data).

##### Real-time-quantitative PCR (RT-qPCR) of gene expression analysis

The molecular expression of the genes *dhn* and *Rd29a* were analyzed. *β-actin* gene was used as an internal standard. Primers used were supplied from Metabion (Germany) (Table S4). The mRNA amount relative to β-actin was measured utilizing the 2^-△△Ct^ approach. Initial denaturation at 95 °C for 5 min, followed by 40 cycles of denaturation at 95 °C for 30 s, annealing at (43–58 °C) for 30s for different template cDNAs and genes specific primers, extension at 72 °C for 30 s, and final extension for 10 min at 72 °C for semi-quantitative reverse transcription-polymerase. Ten µl of PCR products were gathered for semi-quantitative analysis after various cycles prior to actually reaching the plateau phase. By viewing the RT-PCR product on agarose gels after each cycle, the PCR cycles were adjusted to be in the linear range.

##### Analysis of the SYBR green RT-PCR results

The Strata gene MX3005P software determined amplification curves and Ct values. Primers were utilized in a 25- µl reaction containing 12.5 µl of the 2x QuantiTect SYBR Green PCR Master Mix (Qiagen, Germany, GmbH), 0.25 µl of RevertAid Reverse Transcriptase (200 U/µL) (Thermo Fisher), 0.5 µl of each primer of 20 pmol concentration, 8.25 µl of water, and 3 µl of RNA template. The reaction was performed in one step plus real-time PCR machine. To estimate the gene expression variation on the RNA of the different samples, the Ct of each sample was compared with that of the positive control group according to the “ΔCt” method stated by Yuan et al., [37]. Using the following ratio: (ΔCt = Ct housekeeping - Ct target).

## Results

### Isolation of endophytic fungi from stressed wheat leaves

**Fig. 1:**
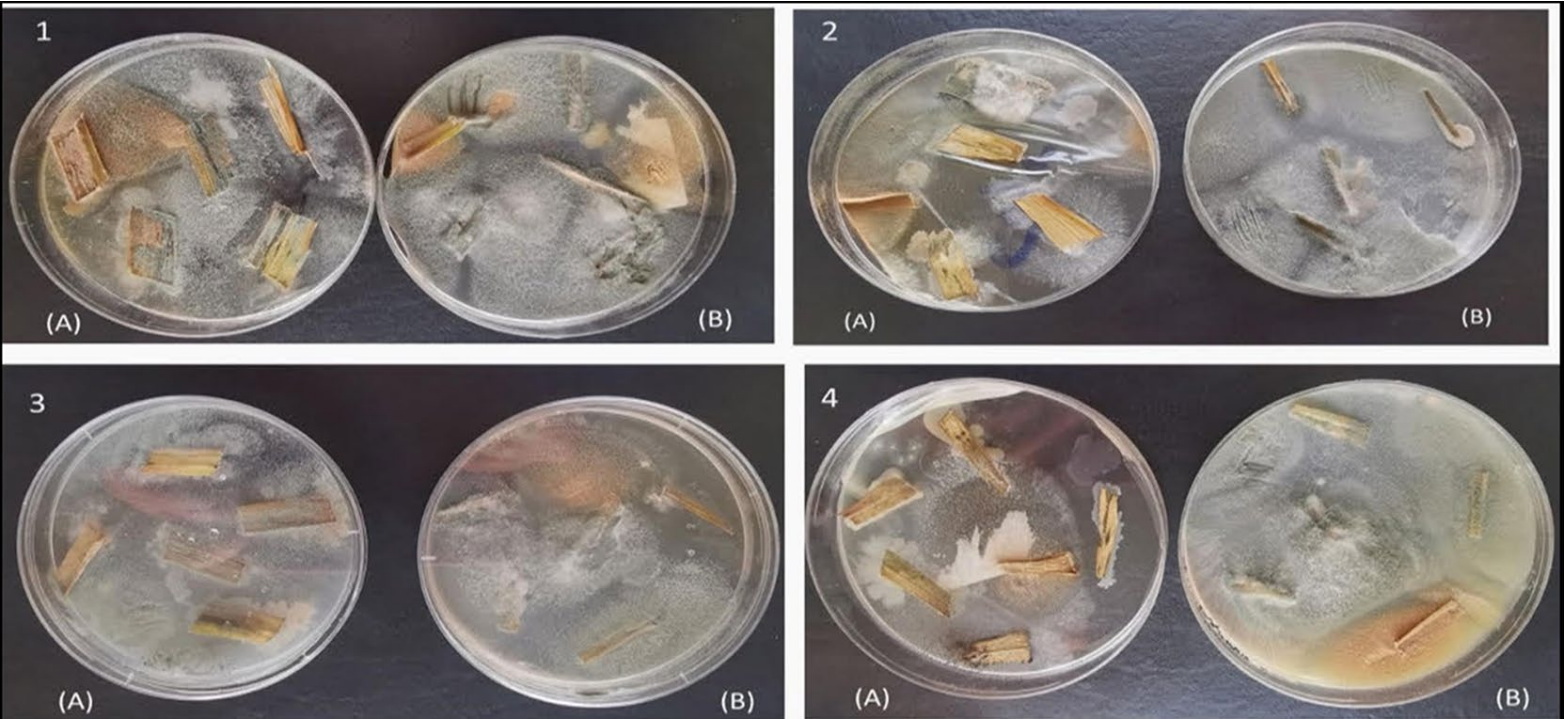
Endophytic fungus isolates from wheat leaves. shown in Pictures 1, 2, 3, and 4. Symbol (**A**) refers to plates of endophyte isolation from control while Symbol (**B**) in all pictures refers to plates of endophyte isolation from stressed plants

**Fig. 2:**
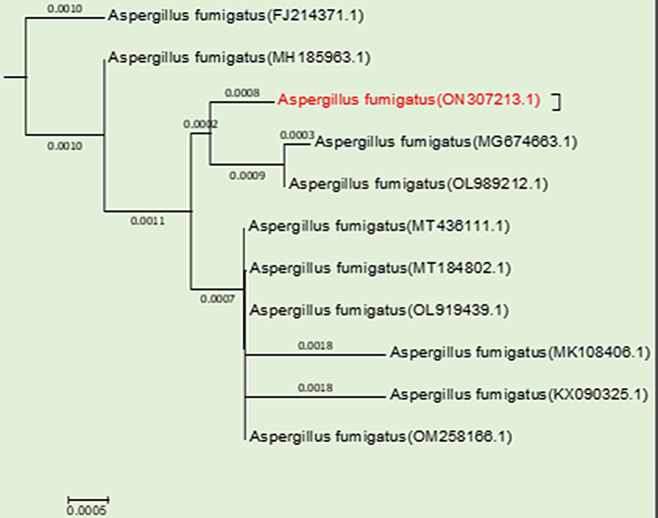
Phylogenetic analysis of *A. fumigatus* (ON307213.1) showing ITS relationship with the ITS sequences of closely related strains retrieved from NCBI GenBank database. Evolutionary analyses were conducted in the MEGA7 program

Endophytic fungal isolates were obtained from the leaves of wheat plants grown on PDA agar medium in response to varying water levels (Fig. 1). These fungal isolates were first identified based on their morphological features using universal keys and were found to belong to the genus *Aspergillus*. The fungal isolates were further identified using both morphological and molecular features. Among the different wheat isolates tested, *Aspergillus fumigatus* was found to be the most frequent at higher drought levels. Microscopic examination revealed non-septate conidiophores with clavate-shaped vesicles. These conidia formed in basipetal chains and arranged uniseriately, green spiked or smooth conidia and septate hyphae with dichotomous branching (Fig. S1). Molecular analysis of the Internal Transcribed Spacer region of rRNA revealed a significant similarity (99–100%) between our isolate and related strains. The NCBI database showed that *a. fumigatus* was deposited on GeneBank with accession # ON307213 (Fig. 2).

### Morphological and physiological responses of different wheat varieties were tested under drought conditions

#### Phenotypic diversity

**Fig. 3:**
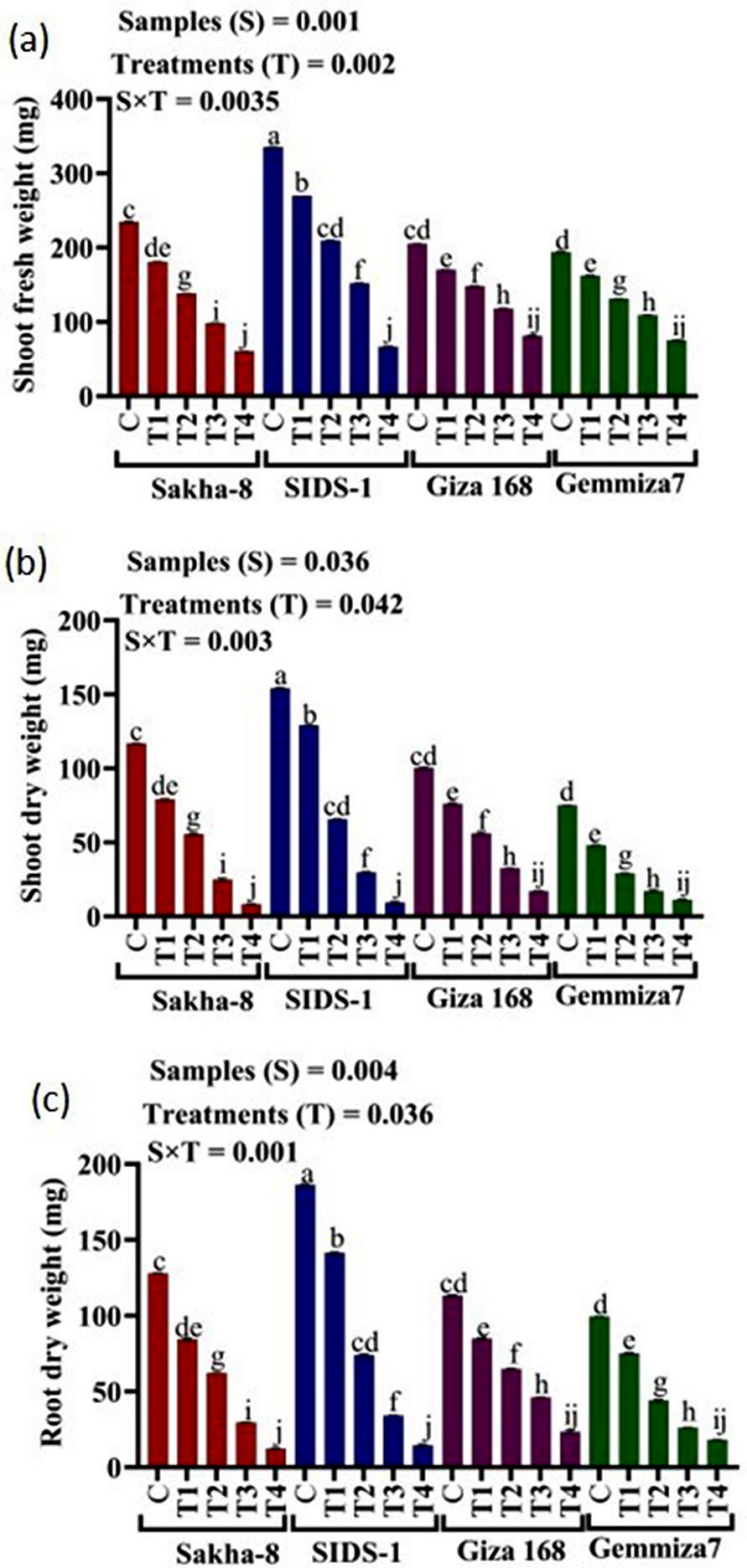
The statistical analysis that shows the fresh and dry weight of shoots and dry weight of roots for different wheat varieties - Gemmiza-7, Sids-1, Giza168, and Sakha-8 - under different levels of drought, as represented in figures (**a**, **b**, and **c**) respectively. The letter “C” represents the control group with 1000 ml of irrigated water, while T1, T2, T3, and T4 refer to groups with decreasing levels of irrigated water − 800 ml, 600 ml, 400 ml, and 200 ml respectively. A P value of S, sample; T, treatments, SxT, the interaction between samples and treatments

**Fig. 4:**
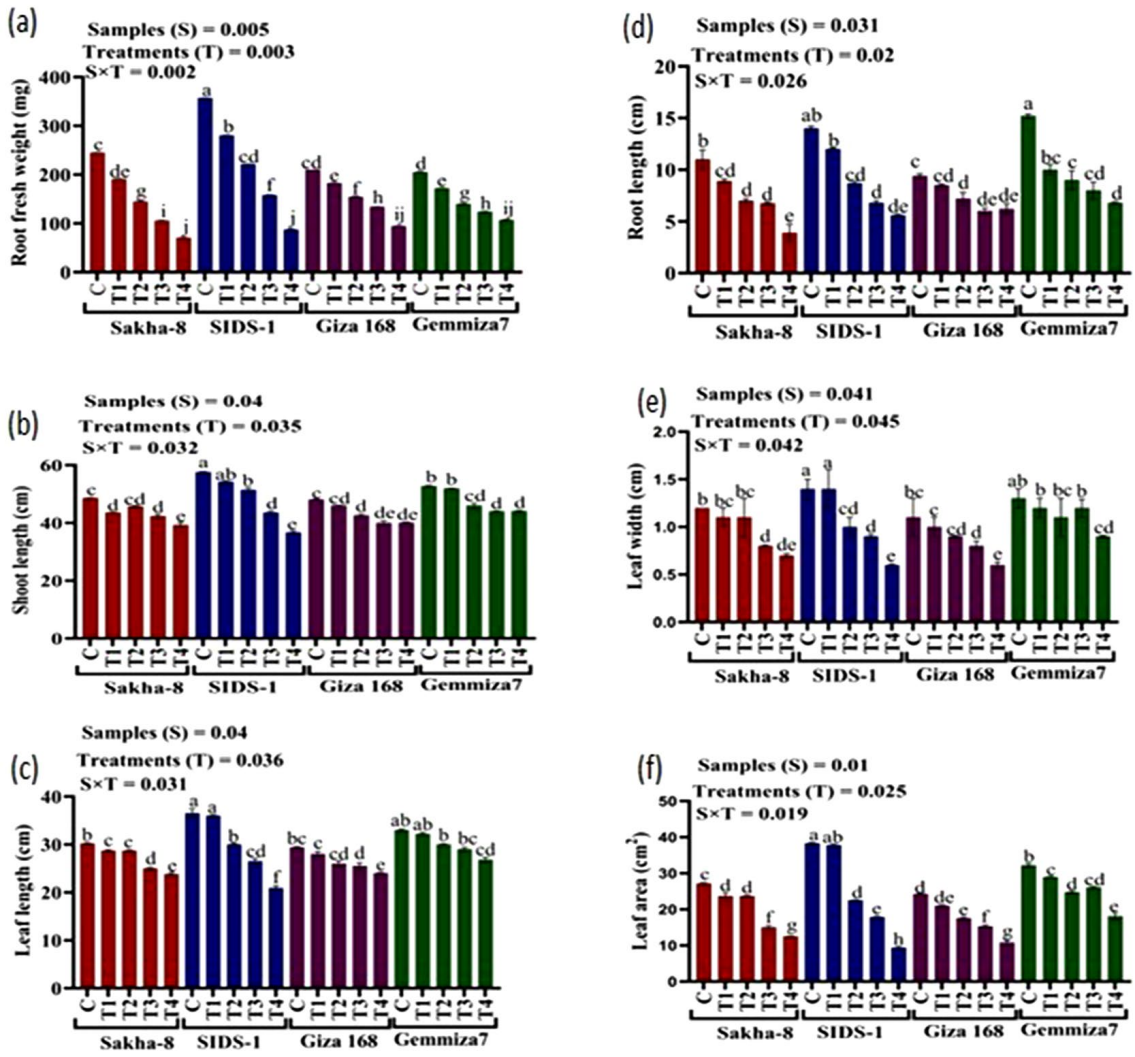
The statistical analysis that shows the root fresh weight, shoot, leaf, and root lengths, leaf width, and leaf area for different wheat varieties - Gemmiza-7, Sids-1, Giza168, and Sakha-8 - under different levels of drought, as represented in figures (**a**, **b**, **c**, **d**, **e**, and **f**) respectively. The letter “C” represents the control group with 1000 ml of irrigated water, while T1, T2, T3, and T4 refer to groups with decreasing levels of irrigated water − 800 ml, 600 ml, 400 ml, and 200 ml respectively. A P value of S, sample; T, treatments, SxT, the interaction between samples and treatments

The study aimed to determine the phenotypic responses of different wheat varieties to varying levels of water stress. The results showed that all wheat varieties exhibited significant morphological changes in response to different levels of drought. Compared to the control, water irrigation ranging from 800 ml to 200 ml produced varying results (Fig. S8). At the highest drought level (200 ml of water irrigation), Giza168 recorded the highest values in shoot fresh weight, shoot dry weight, and root dry weight by 81.2 ± 0.9, 17.12 ± 0, and 23.44 ± 0.9 mg, respectively (Fig. 3a, b and c). On the other hand, Gemmiza-7 recorded the highest values in root fresh weight, shoot length, root length, leaf length, leaf width, and leaf area by 107 ± 0.9 mg, 44.10 ± 0.2, 6.80 ± 0.1, 26.80 ± 0.5 cm, and 18.09 ± 0.9 cm2 respectively (Fig. 4a and f). Meanwhile, Sakha-8 had the highest value in leaf numbers which recorded 5.00 ± 0.1.

**Fig. 5:**
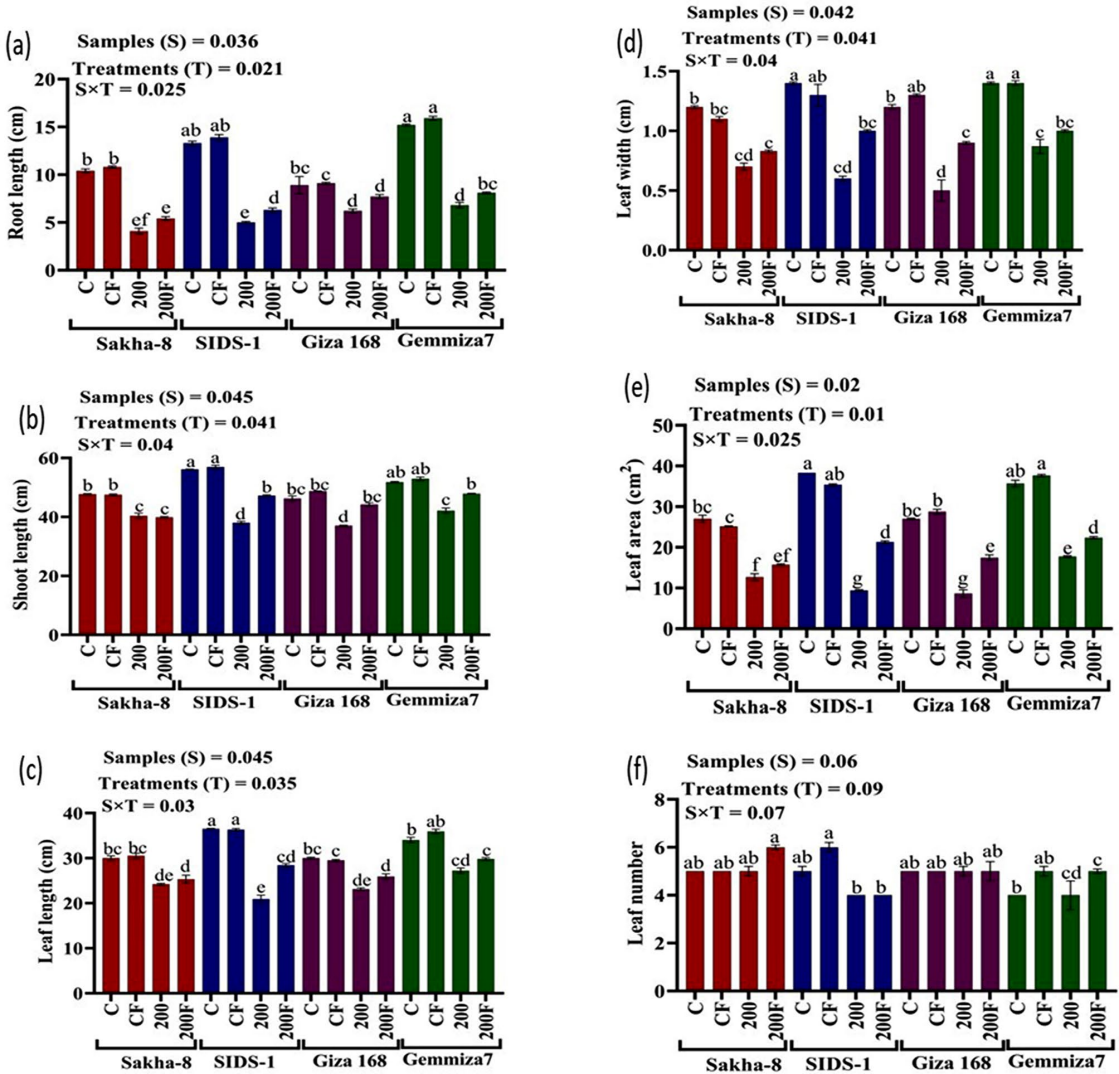
Quantitative phenotypic variations among four wheat varieties. Show the influence of *Aspergillus fumigatus* bio-priming. As a control (non-inoculated with fungus), CF (fungi-inoculated control), 200 (200 ml condition of applied drought, without inoculation), and 200 F (fungi-inoculated 200 ml), where **a** stands for root length, **b** stands for shoot length, **c** stands leaf length, **d** stands for leaf width, **e** stands for leaf area, and **f** stands for leaf number. A P value of S, sample; T, treatments, SxT, the interaction between samples and treatments

**Fig. 6:**
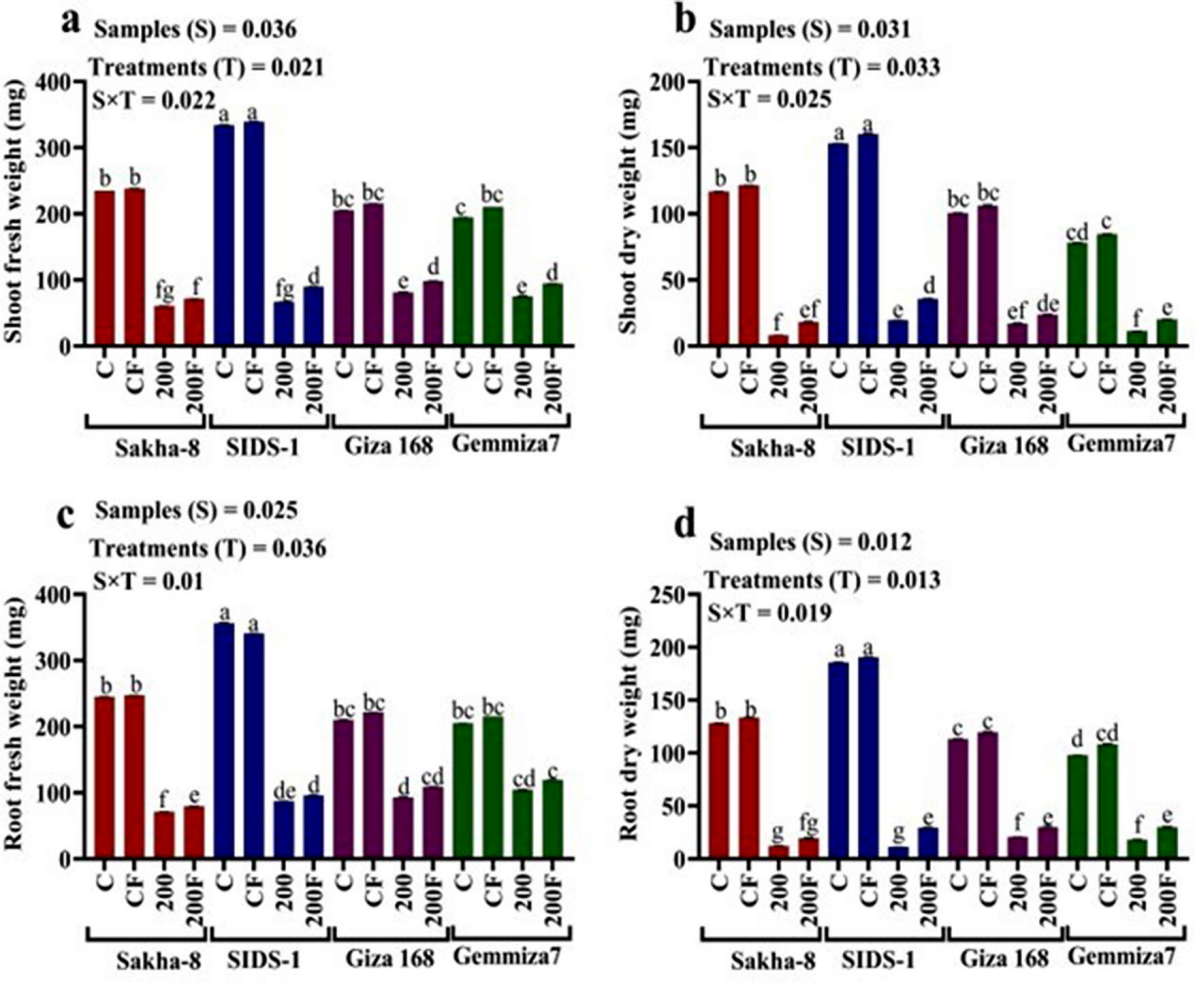
Quantitative phenotypic variations among four wheat varieties, where **a** stands for shoot fresh weight, **b** stands for shoot dry weight, **c** stands for root fresh weight, and **d** stands for root dry weight. Figure shows the influence of *Aspergillus fumigatus* bio-priming. As a control (non-inoculated with fungus), CF (fungi-inoculated control), 200 (200 ml condition of applied drought, without inoculation), and 200 F (fungi-inoculated 200 ml). A P value of S, sample; T, treatments, SxT, the interaction between samples and treatments

Phenotypic differences were observed when comparing bioprimed and un-bioprimed controls unaffected by drought, and bioprimed and un-bioprimed stressed plants irrigated with 200 ml of water. The results are presented in (Figs. 5 and 6). Biopriming had a synergistic effect on the stressed wheat varieties. For instance, in Gemmiza-7, root length, leaf length, shoot length, and root fresh weight significantly increased by 8.1 ± 0.1, 29.8 ± 0.3, 47.9 ± 0.1 cm, and 119.33 ± 0.3 mg, respectively. In Giza168, shoot fresh weight and stem density also increased significantly, recording 98.12 ± 0.5 mg and 829.54 ± 0.6 gm/cm, respectively (Tables S1 and S2). Additionally, leaf width increased significantly in Sids-1, recording a value of 1 ± 0.01 cm (Figs. S4–S7).

In all bread wheat varieties, the mean values of fibrous root number (FR) were higher in four treatments, except in Sakha-8, which was lower than the control in four treatments. After inoculation, the value of FR (11 ± 0.2) became almost equal to more tolerant varieties like Sids-1 (12 ± 0.1). While leaf numbers were slightly lower than controls in four treatments in Gemmiza-7 and Sids-1, the leaf number ratio slightly increased in bio-primed stressed Sakha-8 by a value of 6 ± 0.1.

Most of the twelve phenotypic parameters slightly increased when comparing the inoculated control Sakha-8 to the non-inoculated control Sakha-8 (Table S3). Biological inoculation helps stressed wheat varieties to alleviate drought stress, resulting in a noticeable increase in shoot fresh weight (SFW) from 66.64 ± 0.2 to 89.56 ± 0.6 mg in Sids-1, 81 ± 0.9 to 98.12 ± 0.5 mg in Giza 168, and 75.15 ± 0.9 to 94.04 ± 0.5 mg in Gemmiza-7. In contrast, *A. fumigatus* inoculation increases shoot dry weight in stressed wheat varieties by 9.92 mg in Sakha-8, 16.1 mg in Sids-1, 6.78 mg in Giza168, and 18.89 mg in Gemmiza-7 compared to non-inoculated stressed wheat varieties.

After fungal inoculation, there is no significant difference in root dry weight value in stressed varieties, as Sids-1, Giza168, and Gemmiza-7 all recorded the same root dry weight values, 29.22 ± 0.6, 29.96 ± 0.9, and 29.93 ± 0.8 mg, respectively. There are significant differences between the morphological properties of varieties with p-values of 0.036, 0.031, 0.025, and 0.012 for both fresh and dried weight shoots and roots. Additionally, there are significant differences between treatments, and fungus increase shoot dry weight denotes a reduction in the amount of water evaporated from tissue.

Furthermore, there is no statistically significant difference in shoot length before and after control biopriming, as the values are similar. When the amount of irrigated water is reduced, the plant produces more fibrous roots in search of water. However, biological bio-priming reduces the number of fibrous roots, confirming the synergistic effect of fungal biopriming in stress reduction. Inoculation with endophytic fungi increases root length, implying that if the plant is subjected to abiotic stress, it can develop deeper roots to access water more effectively.

### Physiological and biochemical results

#### Total phenolic content (TPC)

**Fig. 7:**
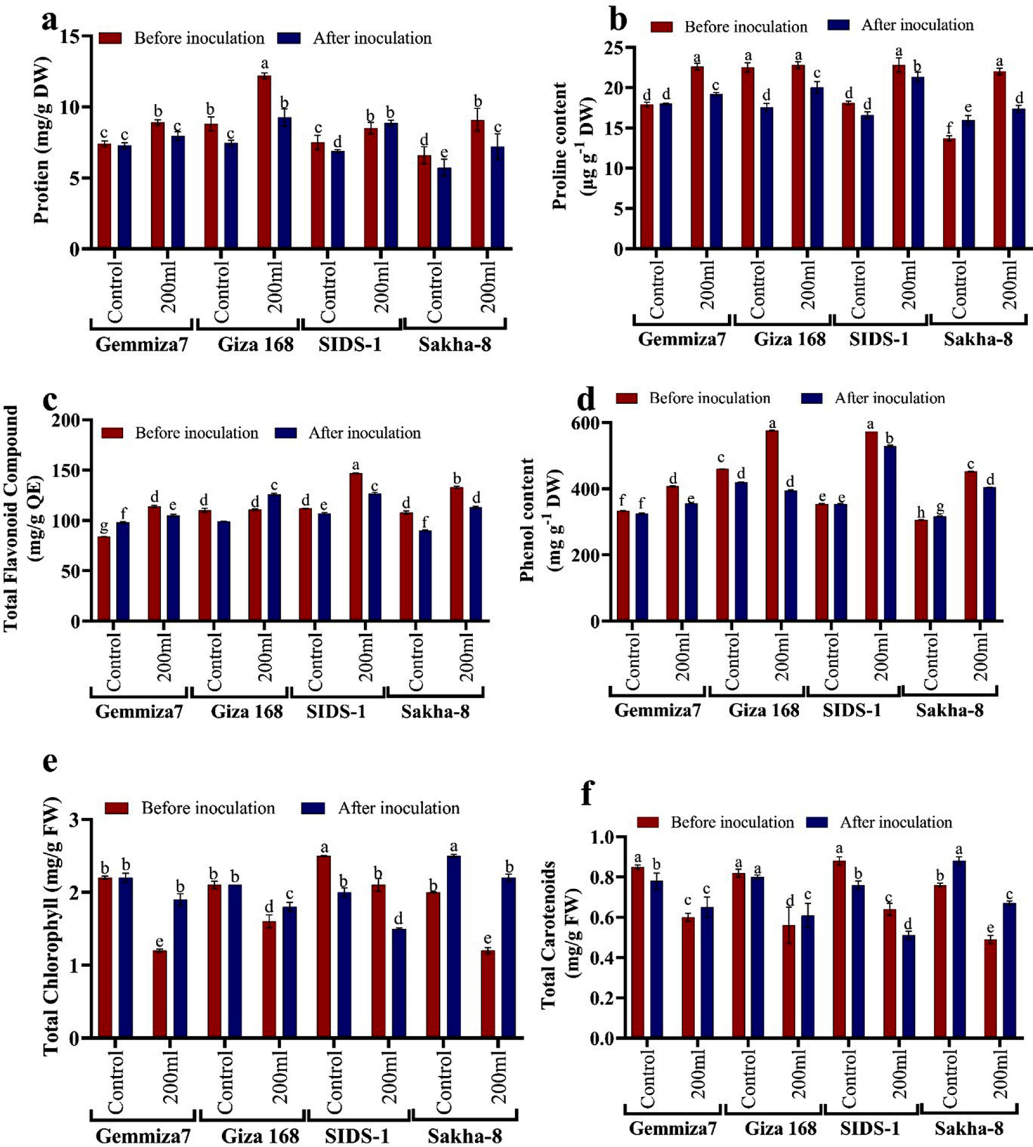
Biochemical assays of non-enzymatic compounds among four wheat varieties *Triticum aestevium* varieties: shakha8, sids-1, Giza168, and Gemmiza7 which shows the influence of *Aspergillus fumigatus* bio-priming. **a** refers to Protein content, **b**. refers to Proline content, **c** refers to Total flavonoid’s compounds, **d** refers to Total phenol content, **e** refers to Total chlorophyll, and **f** refer to Total carotenoids

The study found that the wheat variety Giza-168 showed the highest resistance to drought (200 ml of irrigation) compared to other wheat varieties, with a value of 576.2 ± 1.8 mg/g GAE compared to the control which recorded 460.3 ± 0.5 mg/g GAE. However, when Giza-168 was bio-primed with *Aspergillus fumigatus*, the TPC (Total Phenolic Content) decreased to 395.24 ± 1.8 mg/g GAE. Similarly, when the control was bio-primed, the TPC decreased to 419.84 ± 1.5 mg/g GAE. Fungal biopriming significantly reduced the maximum stress level (200 ml of water irrigation) on other examined wheat varieties. For instance, the TPC of Sakha-8 decreased from 452.4 ± 1.2 to 404.76 ± 0.4 mg/g GAE after biopriming, while that of Sids-1 decreased from 573.0 ± 0.0 to 529.37 ± 2.8 mg/g GAE, and that of Gemmiza-7 decreased from 407.1 ± 2.1 to 356.35 ± 0.5 mg/g GAE. It is worth mentioning that Gemmiza-7 was considered the most tolerant wheat variety in this study (Fig. 7d).

#### Total flavonoid content (TFC)

The total flavonoid content (TFC) decreased in all wheat varieties except Gemmiza-7 after control inoculation with *A. fumigatus*, when compared to the non-inoculated control. However, in Gemmiza-7, the TFC value increased after control inoculation to 98.10 ± 0.9 mg/g QE, compared to the non-inoculated control of 84.1 ± 0.2 mg/g QE. Similarly, when the wheat varieties were subjected to 200 ml of water irrigation, TFC value decreased after inoculation in stressed varieties except in Giza 168, where TFC value increased after inoculation to 126.10 ± 0.9 mg/g QE compared to non-inoculated stressed 111.0 ± 0.6 mg/g QE (Fig. 7c).

#### Proline content

Figure 7b demonstrated that reducing water requirements to a minimum of 20% increased proline content in all wheat varieties compared to control. Inoculation by *A. fumigatus* in stressed wheat seedlings led to reduced proline content of 19.20 ± 0.2, 20.04 ± 0.7, 21.33 ± 0.6, and 17.39 ± 0.4 mM compared to 22.6 ± 0.0, 22.8 ± 0.4, 22.8 ± 0.9 and 22.0 ± 0.4 mM in Gemmiza-7, Giza168, Sids1, and Sakha-8, respectively, compared to the non-inoculated stressed wheat seedlings. Proline content increased slightly after control inoculation in Gemmiza-7 to 17.98 ± 0.1 rather than 17.9 ± 0.1 mM. Still, the ratio increased in Sakha-8, where proline content increased in inoculated control to 15.96 ± 0.6 rather than 13.7 ± 0.3 mM in non-inoculated control. The volatility of the scales increases before control inoculation to 22.5 ± 0.6 and 18.1 ± 0.2 mM and decreases after control inoculation to 17.56 ± 0.5 and 16.58 ± 0.4 mM for Giza168 and Sids-1, respectively.

#### Total protein (TP)

The study found that the protein content in wheat seedlings decreased significantly after being bio-primed with *A. fumigatus* in bio-primed control compared to non-bio-primed control. In resistant variety Gemmiza-7, the TP value reduced slightly from 7.4 ± 0.2 to 7.28 ± 0.2 mg/m, while in Giza168, Sakha-8, and Sids-1, the TP reduced significantly from 8.8 ± 0.5 to 7.45 ± 0.2 mg/m, 6.6 ± 0.6 to 5.73 ± 0.6 mg/m, and 7.5 ± 0.5 to 6.89 ± 0.6 mg/m respectively. The total protein content also decreased in bio-primed drought-stressed wheat seedlings that were watered up by 200 ml compared to un-bio-primed stressed plants, except in Sids-1, where the TP value increased in bio-primed stressed plants to 8.87 ± 0.2 mg/m compared to non-bio-primed stressed Sids-1, which was 8.5 ± 0.4 mg/m. The study concluded that biopriming with *A. fumigatus* enhanced protein content in wheat seedlings grown under drought stress (Fig. 7a).

#### Total chlorophyll (chl)

The results showed that drought treatment (20%) before biopriming significantly affected the activities of total chlorophyll as chlorophyll pigment concentration decreased compared with control. After biological biopriming by *a. fumigates* in stressed seedlings, we found that the concentration of chlorophyll increased by values of 1.90.8, 1.8 ± 0.6, and 2.2 ± 0.5 mg/l compared to un-bio primed stressed seedlings, where chlorophyll concentration 1.2 ± 0.2,1.6 ± 0.9, and 1.2 ± 0.4 mg/l in Gemmiza-7, Giza168, and Sakha-8, respectively. There was a difference in total chlorophyll value in Sids-1 after biological biopriming, as it decreased to 1.5 ± 0.1 mg/l from 2.1 ± 0.9 mg/l before biopriming. There are no significant differences in total chlorophyll value between primed and unprimed controls, except in the sensitive variety Sakha-8, where biological inoculation increased chlorophyll concentration to 2.5 ± 0.2 mg/l rather than 2 ± 0.6 mg/l before control biopriming. This finding supported the efficacy of biopriming in reducing drought stress (Fig. 7e).

#### Total carotenoids content

Abiotic stress, such as drought, reduces the content of accessory pigments like carotenoids. The carotenoid content was calculated considering the effects of *A. fumigatus* priming. Based on the outcomes in Fig. 7f. Total carotenoid values increased after inoculation in stressed seedlings to 0.65 ± 0.05, 0.61 ± 0.06, and 0.67 ± 0.09 mg/l in Gemmiza-7, Giza168, and Sakha-8, respectively, from 0.60 ± 0.02, 0.56 ± 0.09, and 0.49 ± 0.02 mg/l. Sids-1 behaved the same way as in chlorophyll content, as biopriming decreased carotenoids’ content to 0.51 ± 0.02 from 0.64 ± 0.03 mg/l before biopriming. Except for Sakha-8, where carotenoid value increased after biopriming to 088 ± 0.02 mg/l when compared to unprimed control 0.76 ± 0.01 mg/l, total carotenoid content in non-inoculated control is higher than in inoculated control.

#### DPPH assay

**Fig. 8:**
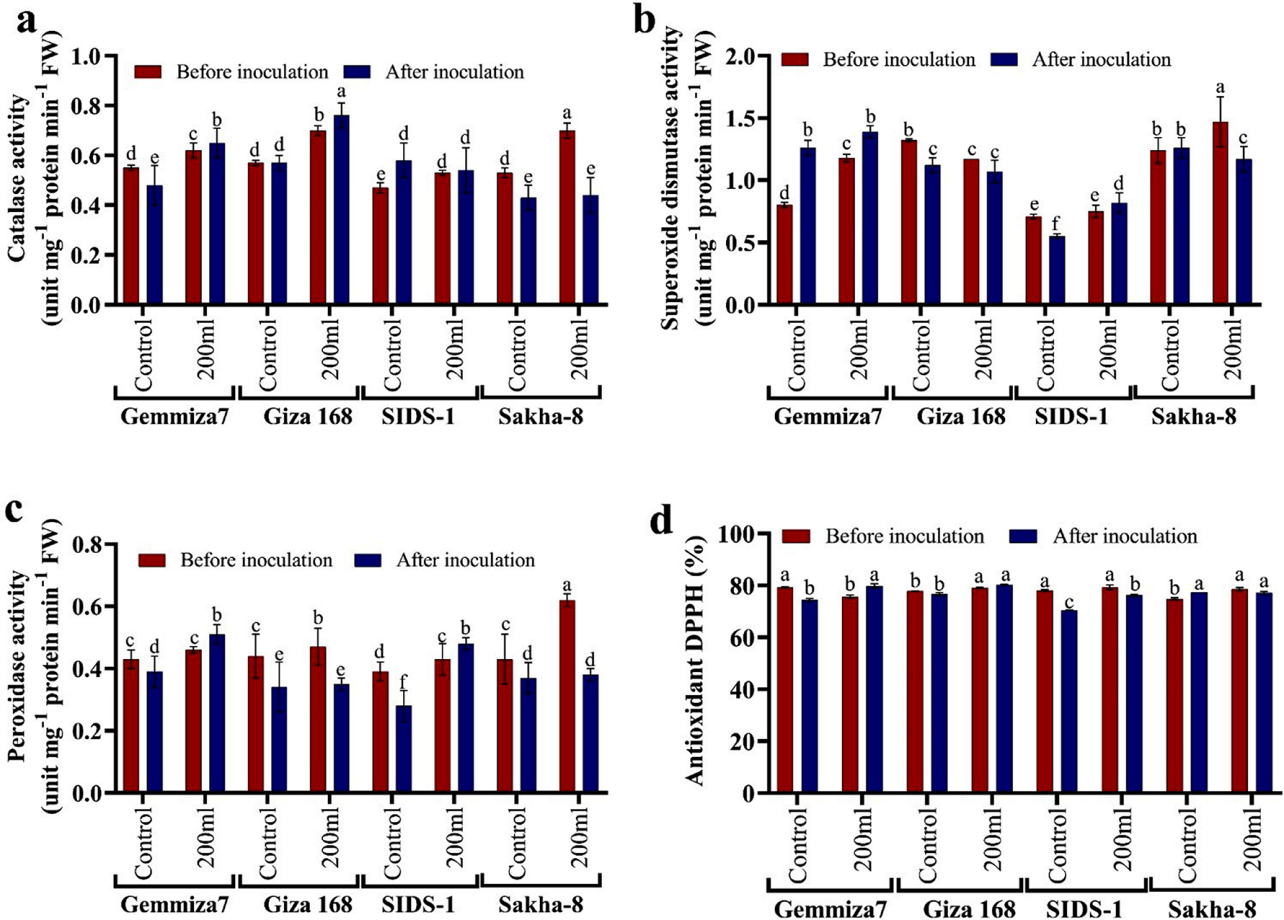
Biochemical assays of enzymatic compounds among four wheat varieties, shows the influence of *Aspergillus fumigatus* bio-priming. **a** refers to Catalase activity, **b**. refers to super oxidase dismutase, **c** refers to Peroxidase activity, and **d** refers to Antioxidant activity

Unlike the control group, when all wheat seedlings were exposed to drought, their defense mechanisms, such as antioxidants, were activated to cope with the stress. In the case of biopriming with *A. fumigatus*, the antioxidant activity increased in bio-primed stressed Gemmiza-7 and Giza168 from 75.73 ± 0.6 and 79.09 ± 0.3 to 79.81 ± 0.9 and 80.290.2% in un-bio primed plants, respectively. For other varieties, Sids-1 and Sakha-8, the antioxidant values decreased in bio-primed stressed plants to 76.21 ± 0.4 and 77.17 ± 0.5 when compared to un-bio-primed stressed Sids-1 and Sakaha-8, where DPPH values were 79.33 ± 0.9% for Sids-1 and 78.61 ± 0.6% for Sakha-8. The effectiveness of fungal inoculation was obvious in sensitive varieties like Sakha-8, where antioxidant activity increased after control biopriming to 77.41 ± 0.0 rather than 74.76 ± 0.5% in un-bio primed control. We also notice that antioxidant activity in most varieties after biopriming in stressed seedlings exceeded the antioxidant activity in normal control seedlings without any stress, confirming the synergistic effect of biological biopriming (Fig. 8d).

#### Catalase (CAT)

Figure 8a depicts catalase activity in four *Triticum aestivium* varieties. The results indicated that a 20% drought level in water irrigation significantly impacted the activities of the catalase enzyme, which increased to counteract stress. The effectiveness of fungus inoculation resulted in increased catalase activity in inoculated stressed varieties except Sakha-8 when compared to the control, confirming the synergistic effect of *Aspergillus fumigatus* in mitigating drought stress. Catalase enzyme value increased in inoculated stressed wheat seedlings to 0.65 ± 0.06, 0.76 ± 0.05, and 0.54 ± 0.09 U/min/g from 0.62 ± 0.03, 0.65 ± 0.06, and 0.53 ± 0.01 U/min/g in non-inoculated stressed Gemmiza-7, Giza168, and Sids-1, respectively. Before inoculation, Sakha-8 had a high CAT activity of 0.70 ± 0.03 U/min/g when compared to inoculated, stressed Sakha-8 0.44 ± 0.07 U/min/g. CAT activity is constant in inoculated and non-inoculated control Giza168 but decreases after control inoculation in both Gemmiza-7 and Sakha-8 to 0.48 ± 0.08 and 0.43 ± 0.05 U/min/g, respectively, compared to non-inoculated control. Only after control inoculation did CAT activity increase to 0.58 ± 0.07 U/min/g from 0.47 ± 0.02 U/min/g.

#### Peroxidase activity (POD)

It is evident from the results that the chosen maximum drought level of 20% water irrigation significantly increased the activity of the POX enzyme compared to the control. The percentage of POX activity under drought stress showed a remarkable rise. By applying biological biopriming to all stressed wheat seedlings, we found that POD activity increased after biopriming in resistant varieties Gemmiza-7 and Sids-1 to 0.51 ± 0.03 and 0.48 ± 0.02 U/min/g, respectively, compared to non-bio primed controls of 0.46 ± 0.01 and 0.43 ± 0.04 U/min/g. POD activity decreased after biopriming in Giza168 and Sakha-8 compared to non-bio primed stressed seedlings, with values dropping to 0.35 ± 0.02 and 0.38 ± 0.02 U/min/g, respectively, from 0.47 ± 0.06 and 0.62 ± 0.00 U/min/g in non-bio primed Giza168 and Sakha-8. Compared to the non-bio-primed control, POD activity decreased in the bio-primed control. According to the findings, biopriming with *Aspergillus fumigatus* aids wheat plants in coping with stress via both enzymatic and nonenzymatic mechanisms (Fig. 8c).

#### Superoxidase dismutase activity (SOD)

The activities of SOD enzymes increased during drought treatment compared to control plants. However, fungal biopriming on stressed plants reduced SOD activity in Giza168 and Sakha-8 seedlings to 1.07 ± 0.09 and 1.17 ± 0.1 U/min/g, respectively, when compared to non-inoculated Giza168 and Sakha-8 seedlings with SOD values of 1.17 ± 0.00 and 1.47 ± 0.2 U/min/g, respectively. In contrast, biopriming increased SOD activity in stressed Gemmiza-7 and Sids-1 by 1.39 ± 0.05 and 0.82 ± 0.08 U/min/g, respectively, compared to non-biopriming stressed Gemmiza-7 and Sids-1, where SOD activity was 1.18 ± 0.03 and 0.75 ± 0.05 U/min/g, respectively. By comparing SOD activity in control before and after biopriming, we found that SOD activity increased after control biopriming in Gemmiza-7 from 0.8 ± 0.02 to 1.26 ± 0.06 U/min/g in bio primed control Gemmiza-7 and in control Sakha-8 1.24 ± 0.1 to 1.26 ± 0.08 U/min/g in bio primed control Sakha-8. SOD activity decreased after control biopriming compared to non-biopriming control in Giza168 and Sids-1, with SOD in Giza168 decreasing to 1.12 ± 0.06 instead of 1.32 ± 0.01U/min/g and in Sids-1 decreasing to 0.55 ± 0.02 from 0.71 ± 0.02 U/min/g (Fig. 8b).

#### Concentration and purity of RNA

This study aimed to examine the impact of drought stress on the genome structure and its effects on drought tolerance and susceptible wheat varieties. The results showed that in susceptible varieties like Gemmiza-7 and Sids-1, the rate of stable RNA and cDNA increased, while in varieties like Giza168 and Sakha-8, the rate of nucleic acid decreased under stress. By comparing the purity and concentration of nucleic acid before and after inoculation, the researchers found that biopriming by *A. fumigatus* can increase the concentration and purity of nucleic acid, indicating the synergistic effect of biopriming in mitigating abiotic stress. The findings are summarized in Table 2.

**Table 2:** Concentration and purity of RNA by nanodrop in (ng/µl) of different wheat varieties

Serial	Sample name	RNA conc (ng/µl)	RNA purity
1	Gemmiza-7 (non-bio primed control)	64.07	1.64
2	Gemmiza-7 (bio-primed control)	25.36	1.89
3	Gemmiza-7 (200lml) non-bio-primed	61.34	1.61
4	Gemmiza-7 (200lml) bio primed	34.22	1.57
5	Giza168 (non-bio primed control)	3.904	1.79
6	Giza168 (bio-primed control)	2.61	1.67
7	Giza168 (200lml) non-bio primed	1.48	1.51
8	Giza168 (200lml) bio primed	3.106	1.72
9	Sids-1 (non-bio primed control)	31.79	1.59
10	Sids-1 (bio primed control)	42.66	1.50
11	Sids-1 (200lml) non-bio primed	39.36	1.69
12	Sids-1 (200lml) bio primed	36.59	1.51
13	Sakha-8 (non- bio primed control)	25.6	1.58
14	Sakha-8 (bio primed control)	2.6	1.68
15	Sakha-8 (200lml) non-bio primed	3.94	1.56
16	Sakha-8 (200lml) bio primed	5.71	1.65

#### RT PCR results

**Fig. 9:**
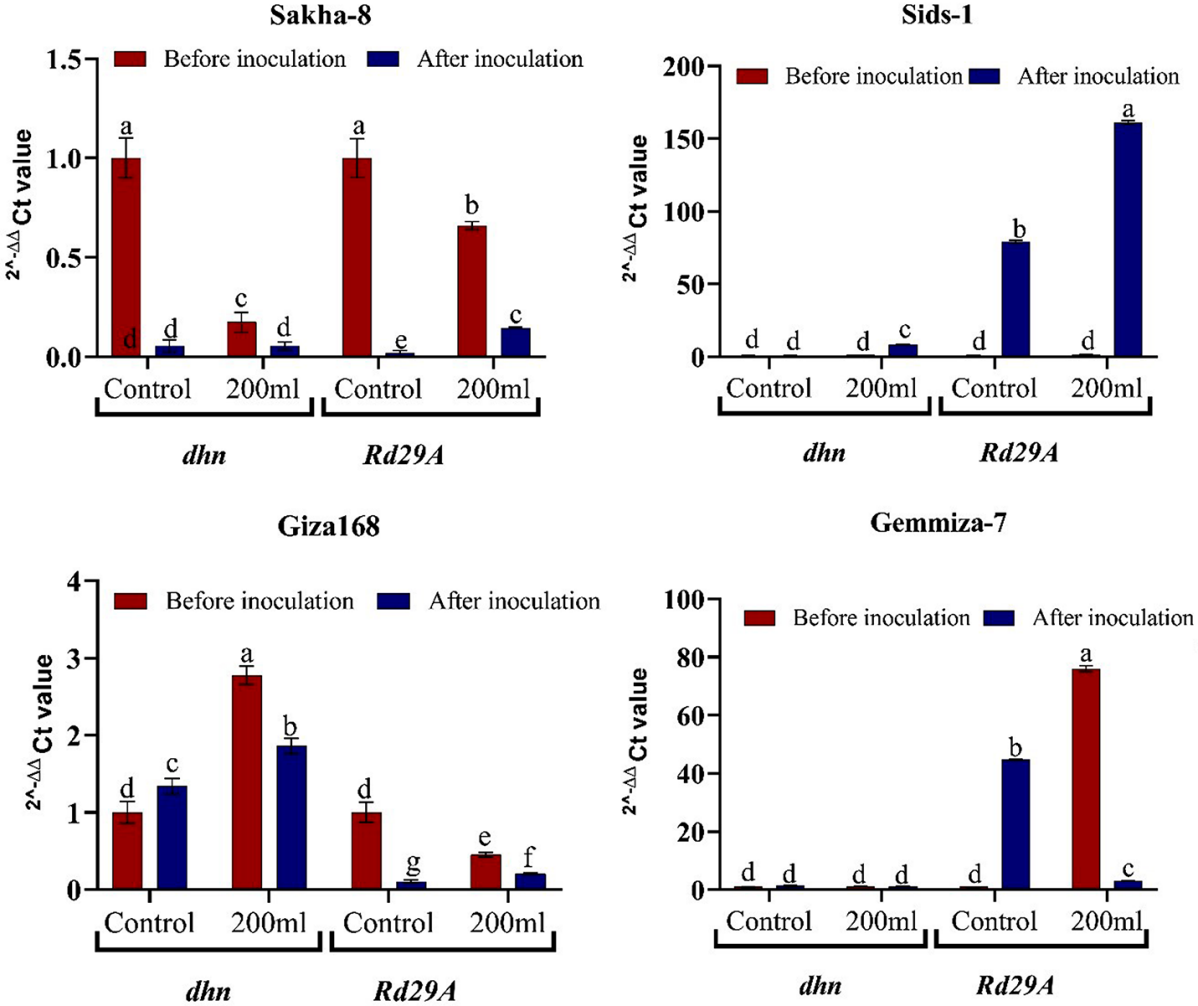
2^-∆∆ Ct value Fold of change values of wheat varieties (Shakha8, Sids-1, Giza168, and Gemmiza7) before and after inoculation by *Aspergillus. fumigatus* illustrate the role of biopriming in upregulation or downregulation of examined genes (*dhn* and *Rd29a*)

We obtained data from the fold of change in (Fig. 9) for *dhn* and *Rd29a* before control biopriming. The 2^-∆∆ Ct values were found to be equal to one. After biopriming, the *dhn* gene was upregulated in all control wheat seedlings but downregulated in Sakha-8. It was then upregulated in both un-bio-primed and bio-primed stressed seedlings, except in Sakha-8, where it was downregulated. Regarding the *Rd29a* gene, we observed increased gene expression in the bio-primed control groups of Gemmiza-7 and Sids-1. On the other hand, there was a decrease in gene expression in Giza168 and Sakha-8. In the un-bio-primed stressed groups of Gemmiza-7 and Sids-1, the expression of *Rd29a* was upregulated, while it was downregulated in Sakha-8 and Giza168. In the bio-primed stressed group, we observed a significant increase in *Rd29a* expression in Sids-1 and a decrease in both Sakha-8 and Giza168 (Table S5).

## Discussion

Drought and water scarcity are among the world’s major problems, and they negatively impact crop production, especially in developing countries. Climate models predict the global surface temperature will increase by 3–5 °C in the next century. This could lead to more frequent occurrences of droughts and floods [38]. Thus, this study aimed to assess the affordability of increasing the physiological and molecular resistance of different cultivars of wheat to drought and water scarcity upon biopriming with endophytic fungi. Wheat morphological traits influenced by water deficit include different leaf characteristics such as shape, area, expansion, size, and cuticle tolerance and root traits including length, density, fresh, and dry weight [39]. The results of our research in (Figs. 3 and 4) suggest that the decline in both root and shoot fresh and dry weight decreases leaf characters in response to abiotic stress. Understanding root-shoot communication is critical for the development of drought-tolerant wheat varieties. Although the roots were directly exposed to the arid settings in the current experiment, it was surprising to discover that it seemed noteworthy that shoot growth was influenced more than root growth. Munns’s research [40] shows many similarities in how plants react to salt and water stress. Abiotic stress decreases a plant’s capacity to absorb water, leading to rapid growth rate reductions and a variety of metabolic changes analogous to those brought on by water stress. The hormonal signal the roots produce is likely to blame for the initial slowdown in shoot growth. There might be salt-specific effects that later affect growth; for example, if too much salt is ingested by the plant, the amount of salt will eventually rise to toxic levels in the older transpiring leaves, causing premature senescence and lowering the plant’s photosynthetic leaf area to a point where growth is no longer possible. Parallel to this, Agarwal and Khan [41] discovered that soybean shoot development was more negatively impacted than root growth. This could be explained by the fact that root growth is typically less vulnerable to saline stress than shoot growth; a higher root/shoot ratio is frequently observed when plants are exposed to drought environments and it observed in Sids-1 and Gemmiza-7, as classified as a tolerant strain compared to others. Different leaf characteristics like form, area, expansion, size, cuticle tolerance, and root characteristics like length, density, fresh weight, and dry weight are among the wheat morphological qualities affected by water deprivation [27]. According to Almaghrabi [42], an investigation has been carried out to evaluate eight wheat (*Triticum aestivum* L.) cultivars, four local cultivars (Madini, Kaseemi, Yamanei, and Tabokei), and four introduced cultivars (Sakha 93, Giza 168, Seds 12, and Masr 1) to drought stress induced by polyethylene glycol (PEG) 6000 at different concentrations. Eight seedling growth characteristics, including shoot length, root length, shoot fresh weight, root fresh weight, and shoot dry, make up the final five germination parameters. A selectable character can be utilized to distinguish between resistant and sensitive cultivars under drought stress in breeding programs based on the results of germination and seedling growth traits, with the exception of root numbers. Fourth, it was discovered that the eight cultivars can be divided into four groups based on their capacity to tolerate stress. An important requirement for wheat’s ultimate productivity is thought to be the capacity to maintain physiological processes under drought circumstances and to recover quickly after re-watering during the vegetative cycle. Modulation of primary metabolism and activation of the antioxidant system has been found to work in concert to protect against dehydration. Enzymatic and non-enzymatic reactive oxygen species (ROS) protection have been demonstrated to be significantly and positively correlated. The extent and duration of drought stress affect how quickly the antioxidant system activates [43]. Contrary to severe drought, which caused a difference in antioxidant enzyme response between tolerant and sensitive wheat varieties, it has been found that mild water stress rarely causes an increase in ROS-scavenging enzyme activity. The buildup of suitable solutes like proline allows wheat plants to regulate their osmotic pressure in response to water shortages. Proline functions as an osmotic regulator, a chaperone, a redox buffer, and a ROS scavenger to preserve proteins and membranes under dehydration stress [44]. El-Saadony et al., [45] confirmed that wheat had a high proline content due to drought stress and lower relative moisture and dry matter generation. They studied the ability of three wheat cultivars, Gemmiza 11, Misr 1, and Giza 171, to withstand drought under three different watering schedules of ten, fifteen, and twenty days. According to the results, cultivar Misr 1 was chosen because it had the best yield attributes coupled with physio-biochemical traits, including chlorophyll, protein, and proline levels that would increase this wheat genotype’s capacity to withstand drought. This study discovered that increased proline levels act as an osmoprotectant in plants, protecting them from drought stress. As shown in (Fig. 7b), when all of the wheat varieties tested were exposed to stress, the proline content increased more than the control. Water shortage conditions limited leaves’ ability to photosynthesize, leading to chloroplast dryness. Drought has been found to negatively influence plant development and metabolism when antioxidants are administered to the leaves [46]. Studies have demonstrated that plants’ responses to drought stress influence nucleic acids, the photosynthetic system, and membrane lipids. As a result of the drought stress, wheat had a high proline and total protein concentration as well as reduced relative moisture and dry matter formation [47]. Drought stress has an impact on total chlorophyll content [48]. These findings supported prior findings that water deficiency circumstances reduced leaf photosynthetic capability, resulting in chloroplast dryness, as shown in (Fig. 7b). Due to the production of reactive oxygen species (ROS) such as oxygen (O_2_) and hydrogen peroxide (H_2_O_2_), such as those caused by heat stress or drought, lipid peroxidation and subsequent chlorophyll degradation can occur. This decrease in chlorophyll concentration is also related to yellowing the leaf’s green tint. There are many ways to induce water stress in plants, ranging from withholding water from them to using chemicals like polyethylene glycol, mannitol, etc. [49]. . El-Hosary et al. [3] discovered that the antioxidant defense system is one of the stress protection systems. As a result, cells of resistant genotypes have established an antioxidant system, such as protective enzymes or antioxidant enzymes like catalase (CAT) and peroxidase (POD), to lower the toxicity of ROS. enzymes like catalase (CAT) and peroxidase (POD) to reduce the toxicity of ROS. Our investigation found that the more tolerant strains had higher levels of antioxidant enzymes, total protein, proline, total flavonoids, and phenolic compounds as a defense mechanism against drought stress. Carotenoids are nonenzymatic antioxidants that play a variety of roles in the tolerance to oxidative stress, including maintaining the photosynthetic apparatus by reacting with lipid peroxidation products to stop chain reactions, scavenging free oxygen radicals, and producing heat as a byproduct. They also prevent singlet oxygen formation through the xanthophyll cycle by reacting with free radicals and generating heat as a byproduct [50]. We discovered that the Sids-1 variety has a higher level of carotenoids. Plants produce more phenolics, aromatic chemicals with benzene rings and one or more hydroxyl groups. When they are under abiotic stress, such as drought. Due to their strong antioxidant properties and notable effects on the protection of different oxidative stressors like salinity, drought, and pathogens, plant polyphenols have attracted growing interest. Plant phenolics and flavonoids have potent biological effects. The group of polyphenolic secondary metabolites known as flavonoids is abundant in plants. Plants produce a wide variety of secondary metabolites called flavonoids that serve a wide range of purposes. Similarly, we observed increased total flavonoid value in sensitive genotypes such as Sakha-8 and Sids-1, indicating susceptibility to stress. Stress-tolerant/resistant species are those that can maintain both total flavonoids and phenolics. These include a variety of activities in defense and signaling between plants and microbes, as well as responsibilities in controlling plant development, pigmentation, and UV protection [51]. Drought alleviation is done through foliar ascorbic acid administration, which influences stomatal closure, nutrient intake, total chlorophyll content, protein synthesis, transpiration, blooming, and photosynthesis. Four wheat varieties (*Triticum aestivum* L.), Mohan Wonder (MW), Kedar (K), Gayatri (GY), and Gandhari (GN), were subjected to drought stress during 3, 6, and 9 days, according to a study by Chakraborty and Pradhan [52]. Drought stress increases lipid peroxidation, proline, phenol, and ascorbate levels, while decreasing total chlorophyll levels. Peroxidase and glutathione reductase activities in cultivars K and GN initially increase, while catalase and superoxide dismutase decrease in MW and GY. K and GN are tolerant, with peroxidase and glutathione reductase playing key roles. The current study also shows that when wheat seedlings are exposed to drought stress, we find a decrease in chlorophyll concentration in all plants, while there is a variation in other enzymatic and non-enzymatic responses in examined wheat seedlings. Drought significantly reduces plant productivity in agricultural fields, leading to increased reactive oxygen species (ROS) and oxidative damage to RNA and DNA. Reducing drought effects can be achieved through appropriate genotypes and agronomic techniques [53]. Our findings, as shown in Table 2, confirmed that drought stress reduced RNA concentrations in stressed Sakha-8 and Giza168 when compared to control.To assess the impact of drought stress on RNA content, 40 old-day peanut seedlings were exposed to varied concentrations of polyethylene glycol-6000 over 24 h in order to assess the effect of the water deficit created by PEG-6000, according to the study of Meher et al., [54]. The findings demonstrated that higher PEG concentrations dramatically lowered RNA content in both leaves and roots, but leaves were more resistant than roots. Chloroplast RNase was elevated, leading to RNA breakdown during water stress and decreased RNA production with greater water stress. In order to protect mRNAs from degradation, ribosomes get crowded on them during water stress conditions. This causes ribosomes to become fragmented, which is another cause of mRNA degradation. One method used to demonstrate the negative effects of drought is the role transcription factors (TFs) play in the control of plant responses to drought stress. To stimulate or inhibit the targeted genes, protein kinases and phosphatases work with particular cis-elements in their promoter regions [55]. The fact that transcription factors (TFs) are important players in the plant responses’ regulation to drought stress is one technique used to illustrate drought’s adverse impact. Protein kinases and phosphatases interact with specific cis-elements in the targeted genes’ promoter region to stimulate or inhibit them [56]. The NAC transcription factors interact with the NAC recognition sequence, which includes a CACG core-DNA binding motif in its promoter, to attenuate downstream drought-inducible early response to dehydration gene transcription, and they are associated with disease resistance and abiotic stress [57]. Dehydrin (*DHN*) genes are members of the Late embryogenesis abundant proteins (LEA) family and are upregulated in wheat in response to stress, such as drought, which causes cell dehydration. They encode *dhn* proteins in wheat, which play a role in protection mechanisms. Many *dhn*s are induced by ABA, so they are also known as RAB proteins (responsive to ABA). Protein denaturation may be prevented by *dhn* proteins binding to the partially dehydrated surface of proteins. They may also be ROS scavengers [58]. Transcription factors like *DREB* (dehydration-responsive element-binding Factor) and *CBF* (Core Binding Factor) control stress-responsive genes. Under osmotic stress, these transcription factors are in play in signal transduction pathways [59]. In the promoter region of drought-tolerance genes like *RD29A*, *DRE*/*CRT* cis-acting elements have been discovered (Dehydration-Resistant 29 A) [60]. Plant genetic engineering and agricultural practices like PGPR and seed biopriming improve plant defense against salt stress, leading to higher crop yields. Bio-priming treats seeds with beneficial microorganisms, improving pre-germination processes and growth. It stimulates phytohormone synthesis, modifies secondary metabolites, adjusts gene expression, and enhances plant resistance to stress [61]. In the research conducted by Abdissa and Bekele [62], four species of *Fusarium* were studied. These species were *F. graminearum*, *F. poae*, *F. avenaceum*, and *F. culmorum*, which were isolated and identified from infected wheat ear samples collected during field assessment. The study concludes that the occurrence of the FHB disease epidemic severely threatens wheat production in the area. Therefore, it is recommended that extensive and intensive surveillance should be carried out across the wheat-growing agroecologies in the country. Farmers should also be trained on the disease’s identification, importance, and management. Integrated disease management is needed as the frontline to reduce the disease problem. Controversial findings on antioxidant and osmolyte fluctuations in PGPR-treated plants highlight the importance of microorganisms like fungi in root development and health, especially under drought stress, in wheat plants [61]. The study of Aizaz et al., [63] identified and analyzed 26 halophilic fungi (endophytic, rhizosphere, and soil) for plant growth-promoting activities from the coastal region of Muscat, Oman. Results demonstrated that compared to the corresponding control plants, the fungal strains MGRF1, MGRF2, GREF2, and TQRF9 reduced 150 mM salt stress and lengthened shoots. However, GREF1 and TQRF9 were seen to increase shoot length in 300 mM stressed plants and in plants that had received salted water (SW) treatment, two strains (GREF2 and TQRF8) also aided plant development and decreased salt stress. Similar patterns were seen in root length as they were in shoot length, with salt stressors such as 150 mM, 300 mM, and saltwater (SW) shortening roots by up to 4%, 7.5%, and 19.5%, respectively. Morsy et al., [64] revealed an elevation in dry shoots and roots compared to non-inoculated plants, with a 3-fold elevation in shoots and a 2-fold increase in roots. Our findings show that *A. fumigatus* increases shoot and root fresh/dry weight as well as shoot and leaf length up to ranges of 10 to 40% of assessed variant phenotypic characteristics. In the current study, *A. fumigatus* bio-primed plants could better ensure the carotenoids and chlorophyll. These findings are in good agreement with a previous study that found that, despite a dose-dependent decline in the contents of both chlorophyll and carotenoids in salt-stressed maize plants after *Trichoderma citrinoviride* biopriming, those concentrations were still significantly higher than those of un-bio primed plants [65]. Another supportive study of our results by Miranda et al., [66] who studied *Zopfiella* strains inoculation in tomato and wheat plants under abiotic stress. Drought stress increased SOD activity in tomato plants only in non-inoculated plants. In contrast, inoculating tomato plants with *Z. erostrata 1* and *Z. erostrata 2* significantly reduced SOD activity under both well-watered and drought stress conditions, when compared to control plants. The activity of APX enzymes was highest in drought-stressed control plants, whereas fungal inoculation resulted in the lowest APX values in drought-stressed plants. Fungal inoculation had the opposite effect on this activity under the well-watered regime. Control wheat plants grown in well-watered conditions had the highest levels of CAT and APX activity, whereas *Z. erostrata 1* and *Z. erostrata 2* inoculated plants had a significant decrease in CAT and APX activity. Tomato plants had nearly the opposite trend as wheat in terms of APX activity. In line with evidence from the literature, the present investigation found that seedlings raised from seeds bio primed with *A. fumigatus* had considerably lower phenol content than seedlings raised from unprimed seeds under drought conditions as shown in (Figs. 7 and 8). Wheat seedlings were seed biopriming with *Trichoderma harzianum*, which raised phenol content [67] The study by Dief et al. [68] found that priming *Triticum aestivum* grains with *Phanerochaete chrysosporium* significantly reduces salt stress, increases growth metrics, and reduces oxidative damage, suggesting *that P. chrysosporium* biopriming could mitigate salinity effects. The chlorophyll and carotenoid levels rose in maize seedlings after seed biopriming before NaCl exposure [69]. Despite the fact that *Trichoderma citrinoviride* biopriming resulted in a dose-dependent decrease in chlorophyll and carotenoids contents in salt-stressed maize plants, their concentrations remained significantly greater than in un-bio-primed plants [65, 68].

The epigenetic modifications that enable the plant to retain some memory of the previous experience even after the stress has subsided also cause long-term changes in gene expression [70]. Through genome imprinting, these heritable epigenetic alterations maintain stress memory and promote memory durability over several generations. It was discovered that priming plants causes them to develop long-term stress memory [71]. This epigenetic stress memory will help plants adapt quickly and recover from damage caused by drought when they are exposed to additional stress [72]. Similar processes, including DNA repair, antioxidant activation, de-novo synthesis of nucleic acids and proteins, and metabolic reactivation, are also triggered in seeds by priming [73].

The recovery from drought stress in tomatoes was accompanied by an upregulation of histone expression, which stimulated DNA replication and returned cell cycle activity [74]. Similarly, seed priming entails the activation of particular enzymes, early DNA replication, and DNA and RNA synthesis [75]. This helps seedlings sprouted from prepped seeds germinate early and uniformly, grow better, and perform better [76]. During seed priming, increased expression of stress-responsive genes and proteins enhances plant abiotic stress sensitivity, allowing them to adapt their metabolism to drought and recovery cycles. This stress memory helps plants change their metabolism and increase cross- and stress tolerance [77]. By focusing on the expression of wheat-resistant genes, we determined that they belong to huge groupings termed transcriptional factors. The part transcription factors (TFs) play in regulating plant responses to drought stress is one way to illustrate the detrimental impacts of drought. *dhn* genes are linked to ABA pathways, and drought stress influences gene expression in plants via ABA-dependent and ABA-independent mechanisms [78]. Figure 9 shows a link between a decrease in plant growth antioxidant levels and an increase in the expression level of *dhn*-resistant wheat seedlings derived from primed grains and a decrease in the expression level of *dhn*-sensitive wheat seedlings. Eight *Trichoderma* strains were tested for their capacity to shield wheat seedlings from extreme water stress. *T. asperellum* (T140). T140 administration response gene downregulation, antioxidant activities, proline content, and low levels of hydrogen peroxide and malondialdehyde increase drought tolerance in wheat seedlings [79]. The relative expression of genes coding for dehydrin-like proteins was elevated in Osmo-primed Spinacia oleracea, and this tendency was also seen there, though it was still more pronounced under drought stress [71]. And these findings support our hypothesis that *A. fumigatus* biopriming reduced the expression of *dhn* in drought sensitive stressed strains like Sakha-8 and Giza168. Kim et al. [80] found that *Enterobacter sp*. EJ01 colonization in Arabidopsis root tissues provides salt stress resistance, enhancing the expression of genes *DREB2b*, *RD29A*, and *RAB18*, and upregulating *P5CS1*, a Pro biosynthesis-related gene, thereby triggering host basal innate immunity and induced systemic resistance. The isolation of *A. fumigatus* from stressed wheat plants, reinoculation with the grain utilizing the bio-priming strategy, and application of stress demonstrate our work’s uniqueness.

## Conclusions

Agriculture is currently being adversely affected by massive climate change and global warming. Because meeting rising need on presence resources is not accessible in today’s world, high-yielding stress-tolerant crops are urgently needed to preserve the balance between food production and rising human demand. Drought deleteriously impacts wheat productivity and grain quality, necessitating the development of drought-tolerant wheat varieties. Wheat varieties are being bred for drought tolerance and to fulfill the demands of an ever-increasing global population using a variety of breeding procedures. Owing to the reduced or increased content of enzymatic and non-enzymatic activities. Microbial seed priming alleviated the drought stress inhibitory impact on the drought-sensitive wheat’s growth and physiological processes. By regulating osmolytes, photosynthetic pigments, and antioxidant enzymes, bio-primed grains were better able to establish into seedlings under drought stress. Based on these findings, one can conclude that the current study paves the way for a new method of mitigating abiotic stress in wheat via seed biopriming with *A. fumigatus*. In this regard, it is critical to investigate the effect of seed bio-priming with *A. fumigatus* on the change of genes associated with stress and seed yield. Another conclusion is that Sids-1, Giza 168, and Gemmiza-7 are the most drought-resistant varieties that resist drought without effort compared to Sakha-8.

### Electronic supplementary material

Below is the link to the electronic supplementary material.

Supplementary Material 1: S1: The morphological features of fungi by displaying conidiophore, strigma vesicle shape, and conidia at 1000x magnification in light microscope photos (1–4). Using a light microscope for morphological identification, we found non-septate conidiophores with clavate-shaped vesicles. Conidia formed basipetal chains and were arranged in an uniseriate manner. Green spiked or smooth conidia. Hyphae septate with dichotomous branching. From microscopic examination, *Aspergillus fumigatus* are characterized. S2: Culture characteristics and molecular identification of *A. fumigatus *isolated from resistant stressed wheat varieties and wheat grains. (A) A culture growing on the surface of PDA agar medium. (B) A 7-day culture growing in broth PDA medium. (C) PCR amplicon of fungal ITS regions for *A. fumigatus* using DNA Ladder 1kb. Molecular analysis of the ITS region of rRNA, revealed significant similarity (99–100%) between our isolate and related strains. NCBI the database giving *A. fumigatus* deposited on Gene bank with accession # ON307213. S3: Experimental design illustrating biopriming treatment of wheat grains, (A) soil sterilization by formalin, (B) holding capacity determination, (C and D) fungal biopriming using carboxymethylcellulose and jute sacks

## Data Availability

The original contributions presented in the study are included in the article, and further inquiries can be directed to the authors.
