# Supplementary material for: Alleviating the drought stress and improving the plant resistance properties of Triticum aestivum via biopriming with aspergillus fumigatus
Source: BMC Plant Biol. 2024 Feb 28;24:150. doi: 10.1186/s12870-024-04840-z (PMC10900732; doi:10.1186/s12870-024-04840-z)
Supplement: Supplementary file 1 — Supplementary Material 1: S1: The morphological features of fungi by displaying conidiophore, strigma vesicle shape, and conidia at 1000x magnification in light microscope photos (1–4). Using a light microscope for morphological identification, we found non-septate conidiophores with clavate-shaped vesicles. Conidia formed basipetal chains and were arranged in an uniseriate manner. Green spiked or smooth conidia. Hyphae septate with dichotomous branching. From microscopic examination, Aspergillus fumigatus are characterized. S2: Culture characteristics and molecular identification of A. fumigatus isolated from resistant stressed wheat varieties and wheat grains. (A) A culture growing on the surface of PDA agar medium. (B) A 7-day culture growing in broth PDA medium. (C) PCR amplicon of fungal ITS regions for A. fumigatus using DNA Ladder 1kb. Molecular analysis of the ITS region of rRNA, revealed significant similarity (99–100%) between our isolate and related strains. NCBI the database giving A. fumigatus deposited on Gene bank with accession # ON307213. S3: Experimental design illustrating biopriming treatment of wheat grains, (A) soil sterilization by formalin, (B) holding capacity determination, (C and D) fungal biopriming using carboxymethylcellulose and jute sacks [file 12870_2024_4840_MOESM1_ESM.pdf]

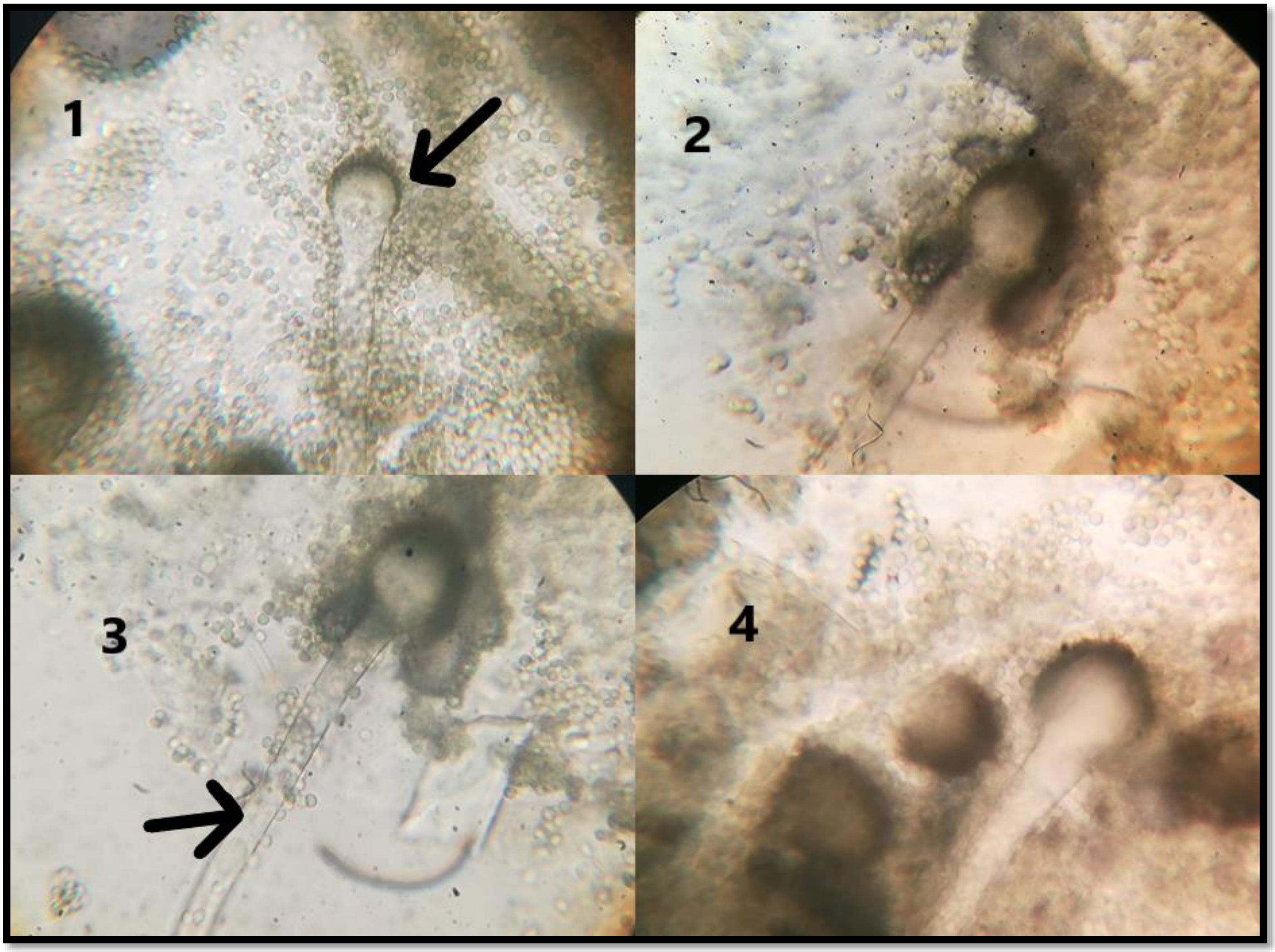

Fig. S1. The morphological features of fungi are displayed by conidiophore, sterigma vesicle shape, and conidia at 1000x magnification in light microscope photos (1-4).

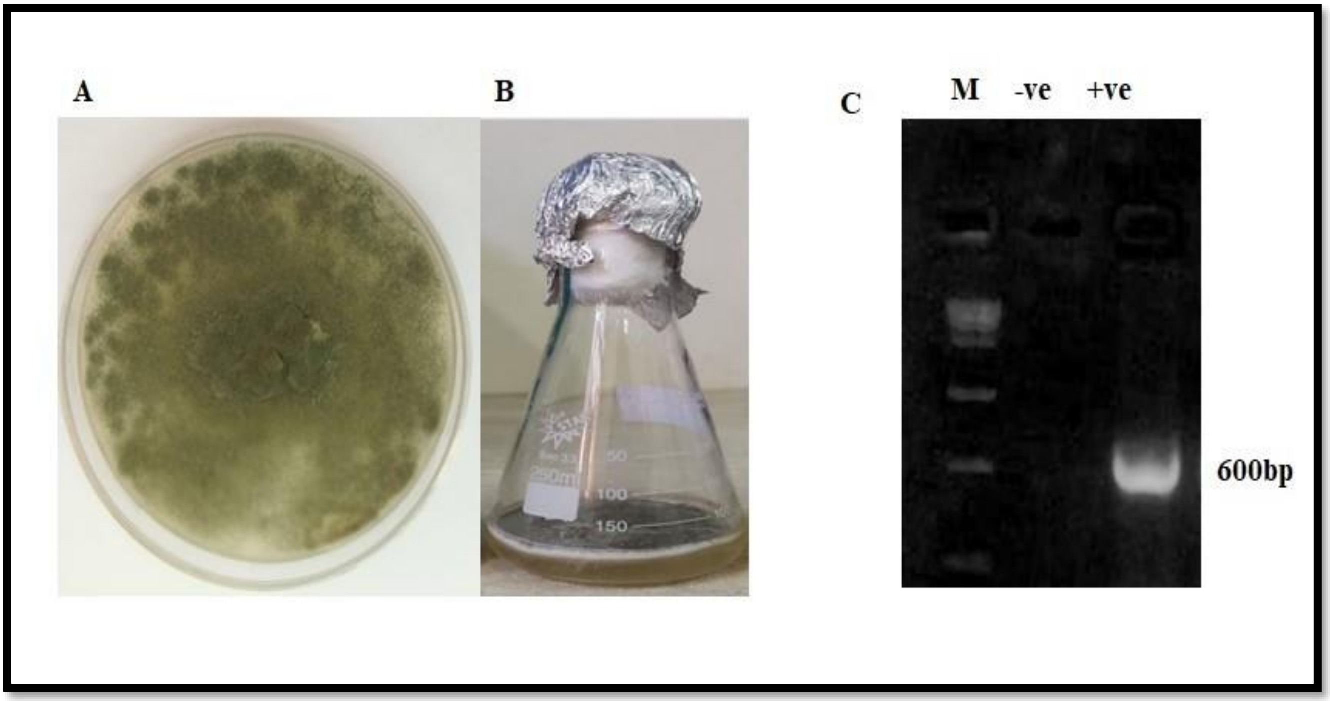

Fig. S2. Culture characteristics and molecular identification of *A. fumigatus* isolated from resistant stressed wheat varieties wheat grains. (A) A culture was growing on the surface of the PDA agar medium. (B) A 7-day culture growing in broth PDA medium. (C) PCR amplicon of fungal ITS regions for *A. fumigatus* using DNA Ladder 1kb.

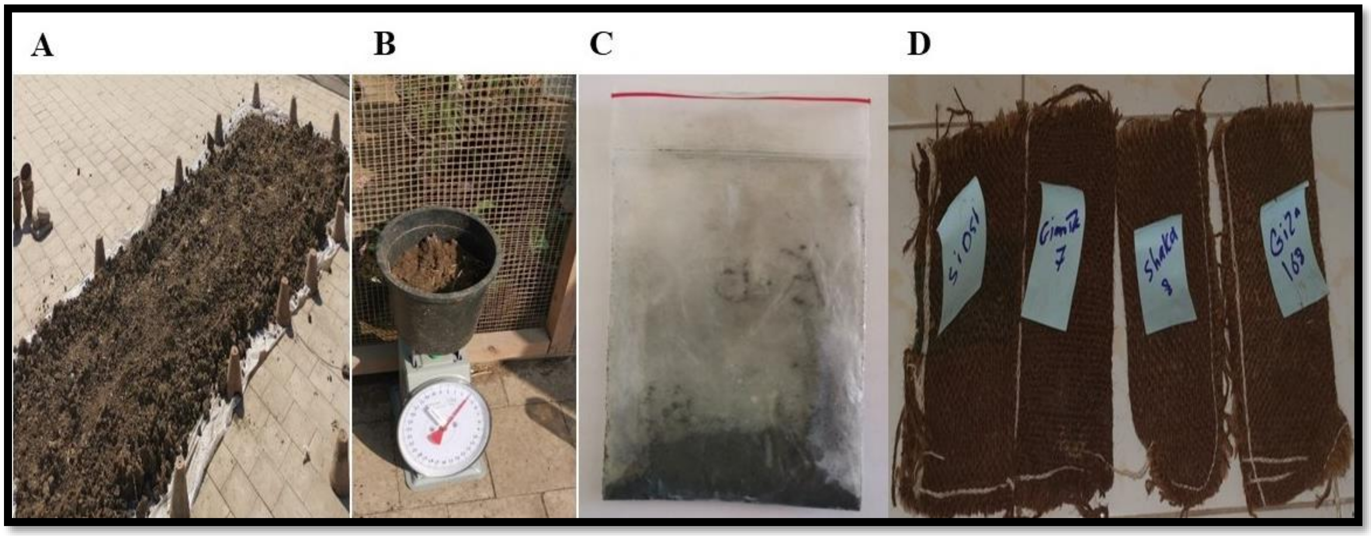

Fig. S3. Experimental design illustrating biopriming treatment of wheat grains, (A) soil sterilization by formalin, (B) holding capacity determination, (C and D) fungal biopriming using carboxy methyl cellulose and jute sacks.

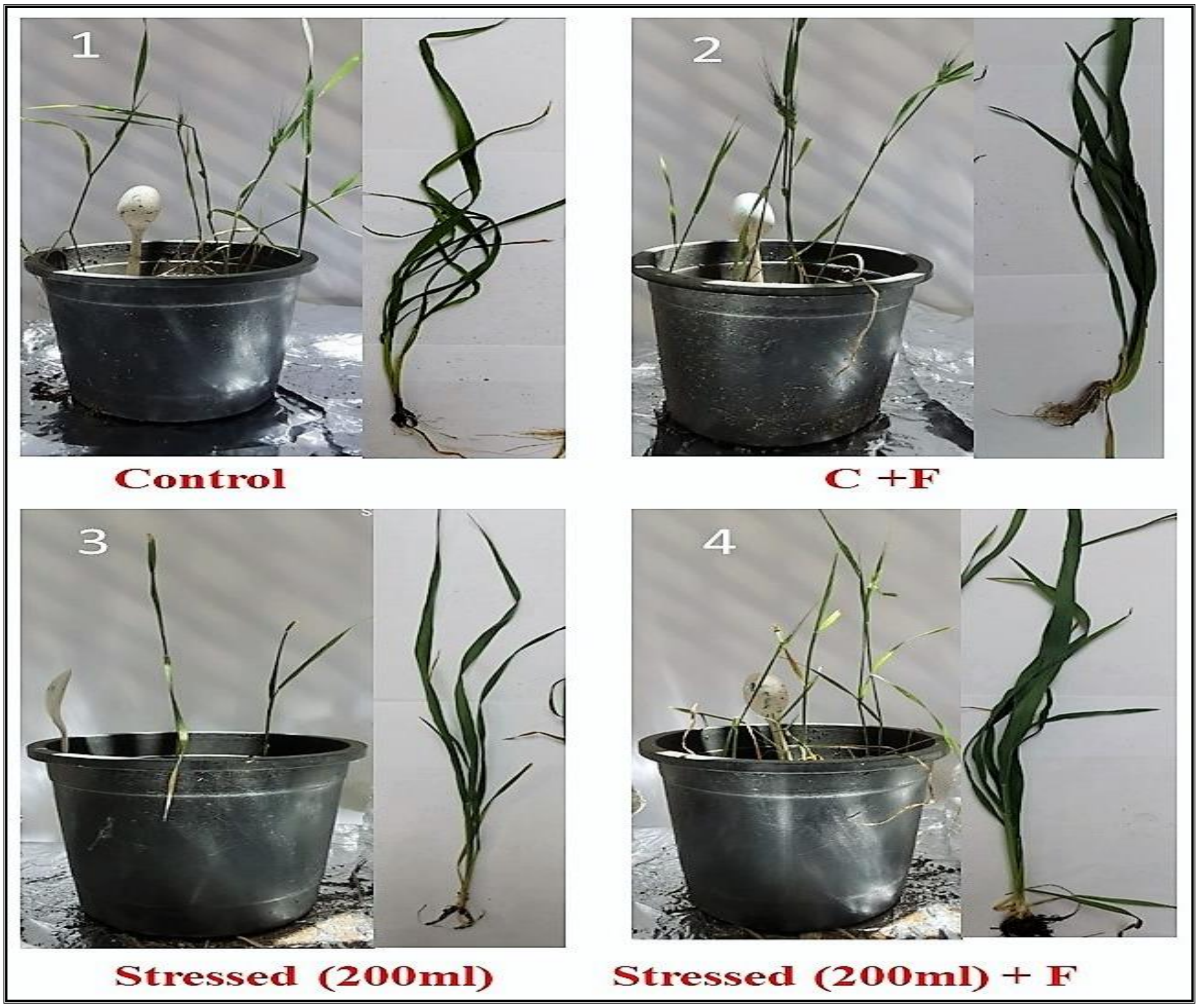

Fig. S4. Morphological effect of fungal biopriming on SIDS-1 before and after biopriming with *Aspergillus fumigatus*. Control Sids-1 referred in picture 1, bio primed Sids-1 referred in pic.2, Stressed Sids-1 before bio-priming represented in pic.3 and bio- primed stressed Sids-1 referred in pic.4.

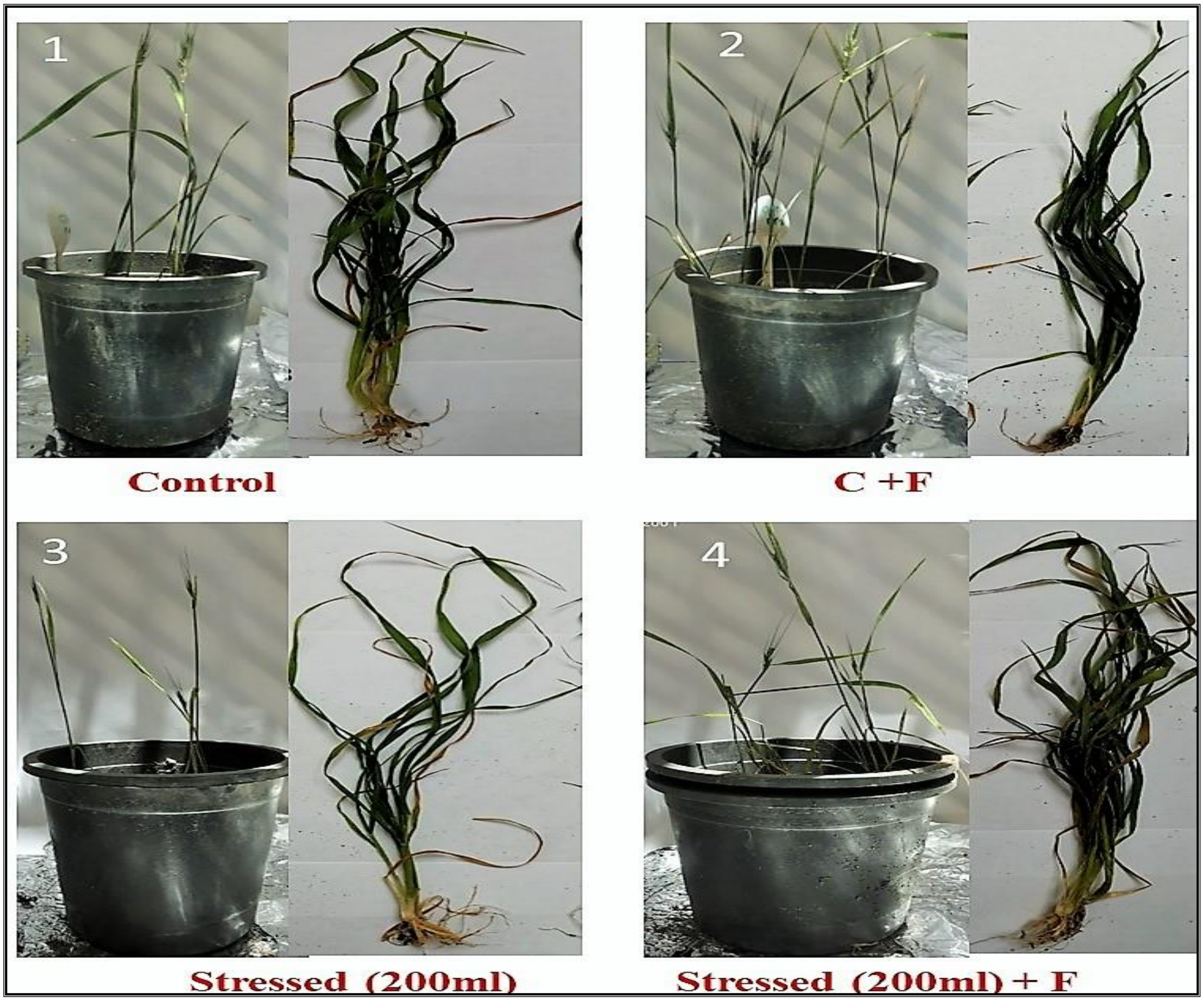

Fig. S5. Morphological effect of fungal biopriming on Giza168 before and after biopriming with *Aspergillus fumigatus*. Control Giza168referred in picture 1, bio-primed Giza168 referred in pic.2, Stressed Giza168 before bio-priming represented in pic.3 and bio-primed stressed Giza168referred in pic.4.

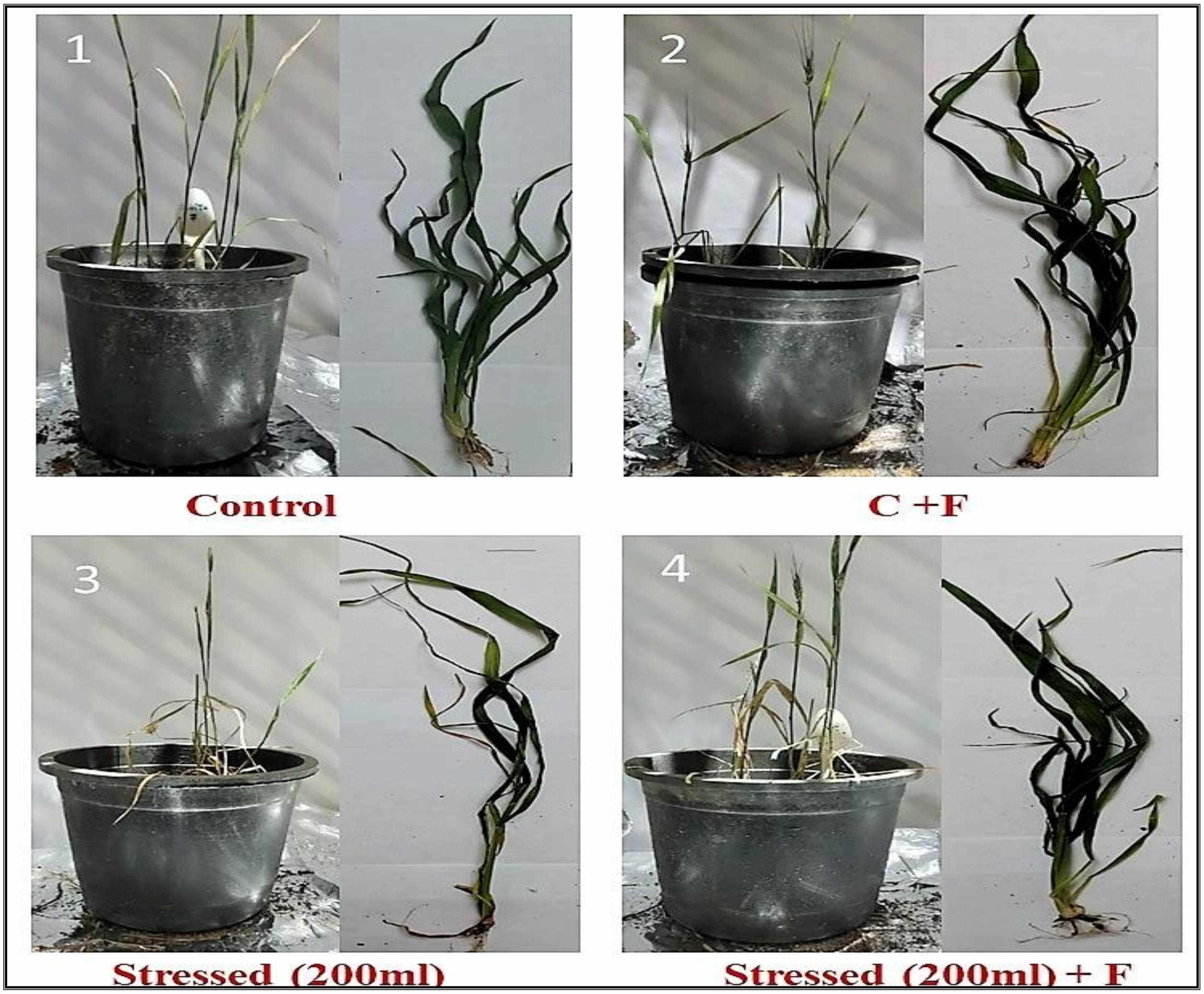

Fig. S6. Morphological effect of fungal biopriming on Gemmiza-7 before and after biopriming with *Aspergillus fumigatus*. Control Gemmiza-7 is referred to in picture 1, and bio-primed Gemmiza-7 is referred to in pic.2, Stressed Gemmiza-7 before bio-priming represented in pic.3 and bio- primed stressed Gemmiza-7referred in pic.4.

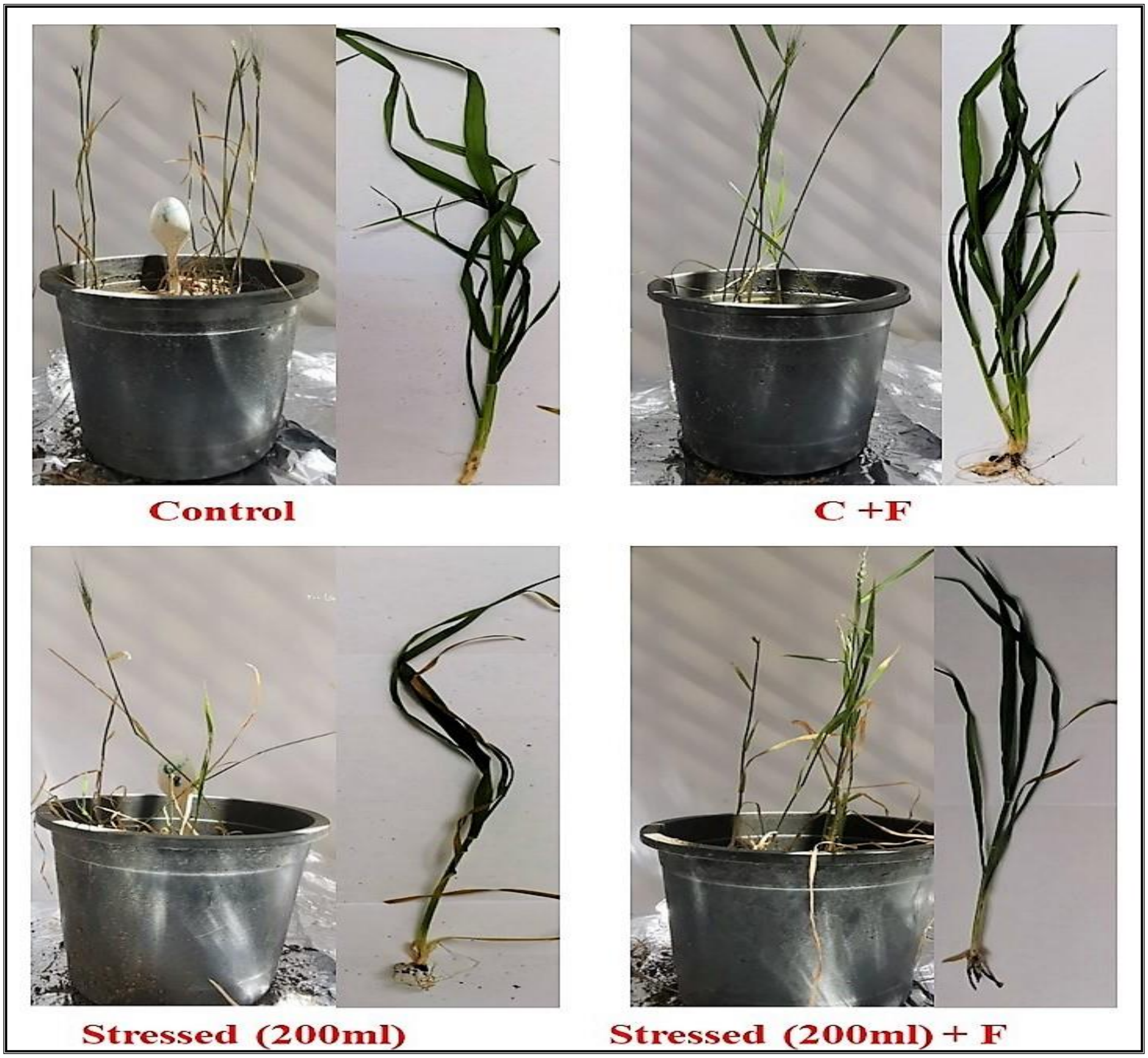

Fig. S7. Morphological effect of fungal biopriming on Sakha-8 before and after biopriming with *Aspergillus fumigatus*. Control Sakha-8referred in picture 1, bio-primed Sakha-8 referred in pic.2, Stressed Sakha-8 before bio-priming is represented in pic.3; bio-primed stressed Sakha-8 is referred to in pic.4.

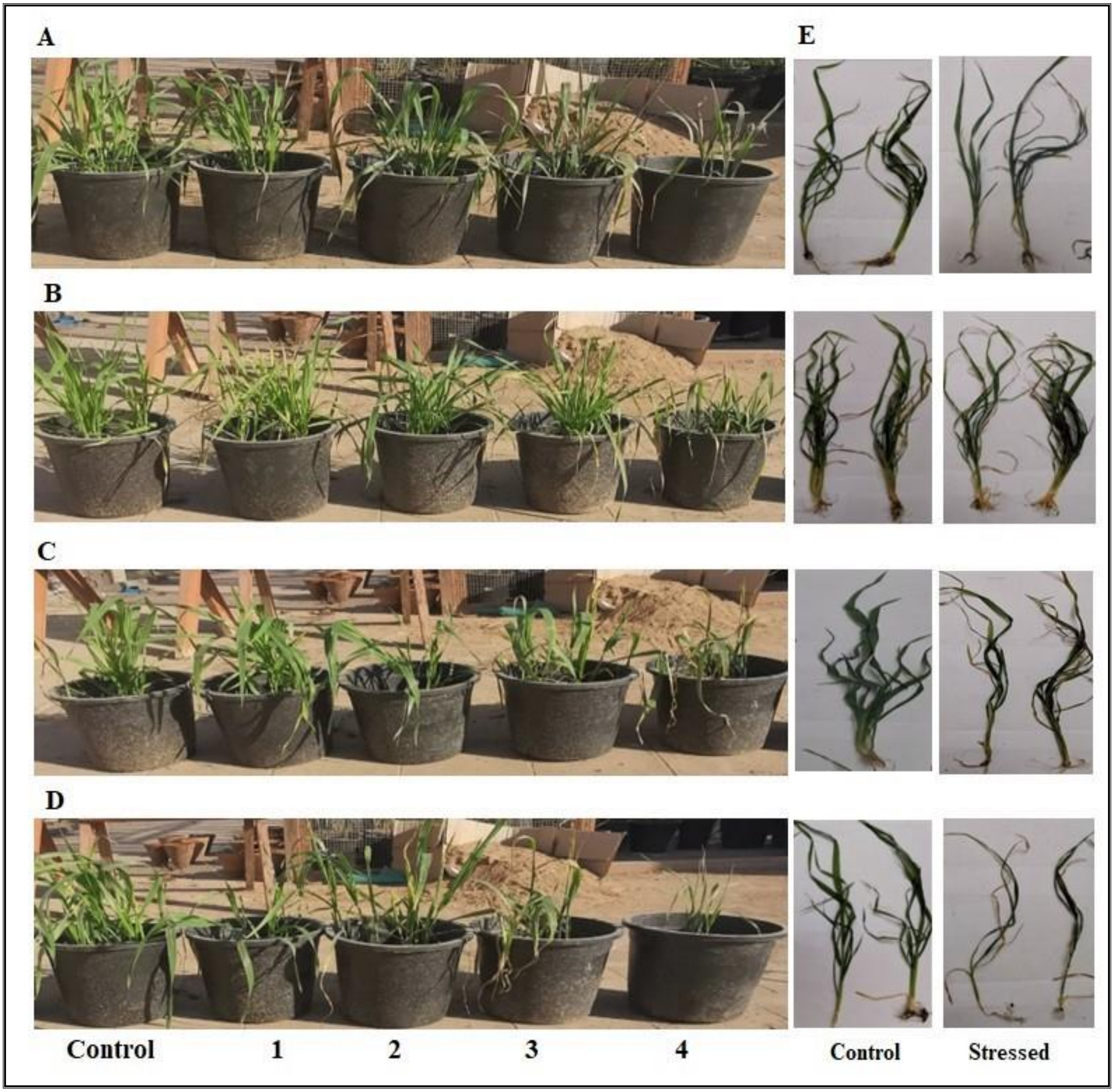

Fig. S8. Morphological effect of drought stress on four distinct wheat varieties and their shoot length SH. L(cm); SIDS-1 (A), Giza 168 (B), Gemmiza-7 (C), and Sakha-8-8 (D). The wheat varieties were cultivated and underwent drought stress by reducing the amount of irrigated water from control (1000 ml), 1: (800 ml), 2: (600 ml), 3: (400 ml), and 4: (200 ml), respectively. In both the control and 200 ml (stressed) irrigated water treatments, the effect of drought stress on the shoot length of the four experimental wheat types was found and quantified (E).

Table S1. Statistical analysis of shoot fresh weight, shoot dry weight, root fresh weight, and root dry weight for different wheat varieties in response to bio-priming with *A. fumigatus*. C stands for control, CF stands for control inoculated with fun-gi, 200 stands for drought stress, and 200F stands for inoculated stressed vari-eties, where n=3, data are presented mean ± SD, lowercase letters in each column indicate significant differences at a probability level of 5%.

N.B. Data are presented in mean ±SD, lowercase (a, b, c, d, …) in the same column, indicating significant difference between the effect of water treatments on wheat varieties (Sids-1, Sakha-8, Giza168, and Gemmiza-7). Uppercase letters in the same raw indicate the significant difference between wheat varieties. A P value of S, sample; T, treatments, SxT, the interaction between samples and treatments.

**Treatment**<br>**Wheat varieties**	**SFW**	**SDW**	**RFW**	**RDW**
**Sakha-8**				
• Control	234.45±0.1b	116.69±0.2b	245±0.2b	127.98±0.2b
• CF	238.15±0.2b	121.34±0.3b	247.02±0.3b	133.45±0.3b
• 200	60.41±0.3fg	8.23±0.1f	70.97±0.3f	12.2±0.1g
• 200F	71.44±0.4f	18.15±0.6ef	79.33±0.4e	19.22±0.9f
**SIDS-1**				
• Control	333.86±0.9a	153.11±0.2a	356.45±0.6a	185.49±0.5a
• CF	339.26±0.1a	160.33±0.6a	340.67±0.3a	190.59±0.2a
• 200	66.64±0.2fg	19.46±0.2e	87.32±0.1de	11.33±0.1g
• 200F	89.56±0.6d	35.56±0.5d	95.86±0.5d	29.22±0.6e
**Giza 168**				
• Control	205±0.1bc	100.4±0.3bc	210.12±0.3bc	113.31±0.3c
• CF	215.09±0.2bc	106.13±0.9bc	221.37±0.2bc	119.73±0.9c
• 200	81±0.9e	16.98±0.5ef	93.11±0.9d	20.66±0.1f
• 200F	98.12±0.5d	23.76±0.3de	108.98±0.1cd	29.96±0.9e
**Gemmiza7**				
• Control	194.45±0.2c	78±0.2cd	204.77±0.2bc	98±0.1d
• CF	210.13±0.1bc	84.45±0.4c	215.14±0.1bc	108.31±0.5cd
• 200	75.15±0.9e	11.27±0.2f	104.03±0.9cd	18.12±0.3f
• 200F	94.04±0.5d	20.22±0.1e	119.33±0.3c	29.93±0.8e
P value				
• *Samples (S)*	0.036	0.031	0.025	0.012
• *Treatments (T)*	0.021	0.033	0.036	0.013
• *S×T*	0.022	0.025	0.01	0.019

Table S2. Statistical analysis of Root length, Shoot length, Fibrous root number, and Stem density for different wheat varieties in response to bio-priming with *A. fumigatus*. C stands for control, CF stands for control inoculated with fungi, 200 stands for drought stress, and 200F stands for inoculated stressed varieties, where n=3, data are presented mean ± SD, lowercase letters in each column indicate significant differences at a probability level of 5%.

N.B. Data are presented in mean ±SD, lowercase (a, b, c, d, …) in the same column, indicating significant difference between the effect of water treatments on wheat varieties (Sids-1, Sakha-8, Giza168, and Gemmiza-7). Uppercase letters in the same raw indicate the significant difference between wheat varieties. A P value of S, sample; T, treatments, SxT, the interaction between samples and treatments.

**Treatment**<br>**Wheat varieties**	**Shoot L.**	**Root L.**	**Fibrous R.**	**Stem D**
**Sakha-8**				
• Control	47.7±0.2b	10.4±.2b	9±0.2d	2446.33±1.2bc
• CF	47.5±0.3b	10.8±0.1b	8±0.3e	2570.94±0.3b
• 200	40.4±0.9c	4.1±0.3ef	12±0.1b	240.47±0.8i
• 200F	39.9±0.2c	5.4±0.2e	11±0.2c	360.47±0.9h
**SIDS-1**				
• Control	56.1±0.1a	13.3±0.2ab	10±0.0cd	2742.78±1.9ab
• CF	56.9±0.6a	13.9±0.3ab	11±0.2c	2819.15±2.1a
• 200	38±0.5d	5±0.1e	14±0.3a	250.23±0.2i
• 200F	47.2±0.3b	6.3±0.2d	12±0.1b	640.78±0.3f
**Giza 168**				
• Control	46.2±0.9bc	8.9±0.9bc	9±0.2d	2173.16±2.1c
• CF	48.7±0.1bc	9.1±0.1c	8±0.6e	2265.32±0.2c
• 200	37±0.2d	6.2±0.2d	13±0.3ab	462.70±0.3g
• 200F	44.2±0.6bc	7.7±0.2d	11±0.1c	829.54±0.6e
**Gemmiza7**				
• Control	51.8±0.2ab	15.2±0.1a	9±0.3d	1467.18±0.9d
• CF	52.9±0.6ab	15.9±0.2a	10±0.2cd	2428.30±0.2bc
• 200	42.1±0.9c	6.8±0.3d	13±0.3ab	356.88±0.1h
• 200F	47.9±0.1b	8.1±0.1bc	13±0.5ab	650.09±0.3f
P value				
Samples (S)	0.045	0.036	0.021	0.001
Treatments (T)	0.041	0.021	0.036	0.001
S×T	0.04	0.025	0.015	0.001

Table S3. Statistical analysis of Leaf length, Leaf number, Leaf width, and Leaf area for wheat varieties in response to bio-priming with *A. fumigatus*. C stands for control, CF stands for control inoculated with fungi, 200 stands for drought stress, and 200F stands for inoculated stressed varieties, where n=3, data are presented mean ± SD, lowercase letters in each column indicate significant differences at a probability level of 5%.

N.B. Data are presented in mean ±SD, lowercase (a, b, c, d, …) in the same column, indicating significant difference between the effect of water treatments on wheat varieties (Sids-1, Sakha-8, Giza168, and Gemmiza-7). Uppercase letters in the same raw indicate the significant difference between wheat varieties. A P value of S, sample; T, treatments, SxT, the interaction between samples and treatments.

Treatment	Leaf L.	leaves No.	Leaf W.	Leave Area
**Wheat varieties**				
**Sakha-8**				
• Control	30±0.5bc	5±0.0ab	1.2±0.01b	27±0.9bc
• CF	30.5±0.6bc	5±0.0ab	1.1±0.02bc	25.16±0.1c
• 200	24.2±0.2de	5±0.2ab	0.7±0.03cd	12.70±0.8f
• 200F	25.3±0.9d	6±0.1a	0.83±0.01c	15.74±0.2ef
**SIDS-1**				
• Control	36.5±0.1a	5±0.2ab	1.4±0.01a	38.32±0.0a
• CF	36.3±0.3a	6±0.2a	1.3±0.09ab	35.39±0.2ab
• 200	20.9±0.9e	4±0.0b	0.6±0.02cd	9.40±0.2g
• 200F	28.4±0.4cd	4±0.0b	1±0.01bc	21.31±0.3d
**Giza 168**				
• Control	30±0.2bc	5±0.0a	1.2±0.02b	27±0.2bc
• CF	29.5±0.2c	5±0.0	1.3±0.01ab	28.76±0.6b
• 200	23.1±0.3de	5±0.2	0.5±0.09d	8.66±0.9g
• 200F	25.9±0.6d	5±0.4	0.9±0.01c	17.45±0.7e
**Gemmiza7**				
• Control	34±0.6b	4±0.0b	1.4±0.01a	35.7±0.8ab
• CF	35.9±0.5ab	5±0.2a	1.4±0.02a	37.65±0.3a
• 200	27.2±0.6cd	4±0.6b	0.87±0.06c	17.74±0.2e
• 200F	29.8±0.3c	5±0.1a	1±0.01bc	22.35±0.3d
P value				

• ***Samples (S)***	**0.045**	**0.06**	**0.042**	**0.02**
• ***Treatments (T)***	**0.035**	**0.09**	**0.041**	**0.01**
• ***S×T***	**0.03**	**0.07**	**0.04**	**0.025**

Table S4. Forward and reverse sequence of primers *dhn* and *β-actin* genes according to (Hassan *et al.*, 2015), and *Rd29A* gene according to (Li *et al.*, 2019).

Target gene	Forward and reverse primers at 5′–3′ sequences	Reference
dhn	**f 5′ATGGAGCACCAGGGGC3′**<br>**r 5′GCAGCTTGTCCTTGATCTTG3**	**Hassan *et al.*, (2015)**
β-actin	**f 5′TCGCTGACCGTATGAGCAAAG3**<br>**r 5′TGTGAACGATTCCTGGACCTG3′**	**Hassan *et al.*, (2015)**
Rd29A	**f 5′ATCACTTGGCTCCACTGTTGTTC3′**<br>**r5′ACAAAACACACATAAACATCCAAAGT3′**	**Li *et al.*, (2019).**

Table S5. 2^-ΔΔ Ct value Fold of change value of wheat varieties before and after inoculation by *A. fumigatus* illustrate the role of biopriming in the upregulation or downregulation of examined genes.

serial	Gene Name	treatment	2^-ΔΔ Ct value Fold of change							
			Wheat varieties							
			Gemmiza-7		Giza168		Sids-1		Sakha-8	
			Before inoculation	After inoculation	Before inoculation	After inoculation	Before inoculation	After inoculation	Before inoculation	After inoculation
1	*dhn*	control	1.002	1.340	1.003	1.341	1.014	1.116	1.0009	0.0538
		200ml	1.109	1.142	2.78	1.86	1.168	8.436	0.175	0.0546
2	*Rd29A*	Control	1.002	44.75	1.002	0.1035	1.0002	79.068	1.0004	0.0181
		200ml	76.045	3.168	0.451	0.2090	1.583	161.08	0.660	0.146
